# Pharmacochemical Studies of Synthesized Coumarin–Isoxazole–Pyridine Hybrids

**DOI:** 10.3390/molecules30071592

**Published:** 2025-04-02

**Authors:** Matina D. Douka, Ioanna M. Sigala, Catherine Gabriel, Eleni Nikolakaki, Dimitra J. Hadjipavlou-Litina, Konstantinos E. Litinas

**Affiliations:** 1 Laboratory of Organic Chemistry, Department of Chemistry, Aristotle University of Thessaloniki, 54124 Thessaloniki, Greece; doukamatina@chem.auth.gr; 2 Laboratory of Biochemistry, Department of Chemistry, Aristotle University of Thessaloniki, 54124 Thessaloniki, Greece; isigala@chem.auth.gr (I.M.S.);; 3 HERACLES Research Center on the Exposome and Health, Center for Interdisciplinary Research and Innovation, Balkan Center, Thermi, 57001 Thessaloniki, Greece; katerinagabriel@cheng.auth.gr; 4 Laboratory of Environmental Engineering, Department of Chemical Engineering, Aristotle University of Thessaloniki, 54124 Thessaloniki, Greece; 5 Laboratory of Pharmaceutical Chemistry, School of Pharmacy, Faculty of Health Sciences, Aristotle University of Thessaloniki, 54124 Thessaloniki, Greece; hadjipav@pharm.auth.gr

**Keywords:** coumarin–isoxazole–pyridine hybrid, 1,3-dipolar cycloaddition reaction, propargyloxycoumarin, propargylaminocoumarin, pyridine aldehyde oxime, phenyliodine(III) diacetate (PIDA), tert-butyl nitrite (TBN), LOX inhibitors

## Abstract

Several new coumarin–isoxazole–pyridine hybrids were synthesized through a 1,3-dipolar cycloaddition reaction of nitrile oxides, prepared in situ from pyridine aldehyde oximes, with propargyloxy- or propargylaminocoumarins in moderate-to-good yields. Synthetic modifications were applied using (diacetoxyiodo)benzene (PIDA) at room temperature, microwave irradiation, or tert-butyl nitrite (TBN) under reflux. Coumarin, isoxazole, and pyridine groups were selected for hybridization in one molecule due to their biological impact to inhibit lipid peroxidation and an enzyme implicated in inflammation. Preliminary in vitro screening tests for lipoxygenase (LOX) inhibition and anti-lipid peroxidation for the new hybrids were performed. A discussion on the structure–activity relationship is presented. Compounds **12b** and **13a** were found to be potent LOX inhibitors with IC_50_ 5 μΜ and 10 μΜ, respectively, while **12b** presented high (90.4%) anti-lipid peroxidation. Furthermore, hybrids **12b** and **13a** exhibited moderate-to-low anticancer activities on HeLa, HT-29, and H1437 cancer cells.

## 1. Introduction

Reactive oxygen species (ROS) are continuously produced in the human body as byproducts of cell metabolism. Some of them are characterized as highly toxic [1]. Their extreme reactivity and the tendency to induce chain reactions lead to pathological processes like inflammation, asthma, and cardiovascular and neurological disorders. Lipid peroxidation is one of the major outcomes of ROS-mediated injury. It directly damages membranes and generates several products that possess neurotoxic activity [1]. ROS exerts toxic effects and directly oxidizes biological macromolecules, such as proteins, nucleic acids, and lipids, further exacerbating the development of inflammatory responses and causing various inflammatory diseases.

Lipoxygenase (LOX) is the key enzyme in leukotriene biosynthesis [2]. Leukotrienes are derived from the biotransformation of arachidonic acid catalyzed by 5-lipoxygenase (5-LOX). They are inflammatory mediators, causing inflammation, cancer, and stroke. LOXs contribute to membrane lipid peroxidation by forming hydroperoxides in the lipid bilayer, whereas cerebral ischemia-reperfusion triggers lipid peroxidation and inflammation. Inhibitors of LOX have attracted attention initially as potential agents for inflammatory disease treatment and for certain types of cardiovascular diseases [2].

Coumarin derivatives of natural or synthetic origin represent a large variety of compounds with diverse biological and pharmacological properties [3,4,5,6,7,8,9,10]. These properties include, amongst others, anti-HIV [11], anticancer [12], antioxidant [13], anti-inflammatory [13,14], anti-Alzheimer [15], antidepressant [16], antibacterial [17], anticonvulsant [18], antitubercular [19], and anticoagulant [20] activities.

The hybrid drug concept is an alternative sophisticated approach of combination therapy applicable in the treatment of complex and multifactorial diseases such as cancer, infectious and inflammatory diseases, and neurological disorders when traditional single-target therapy is not satisfactory [21]. Hybrids that scavenge ROS have emerged as an important approach used to limit inflammatory responses and protect the host against damage [22]. Molecular hybridization is a drug design strategy that combines two or more pharmacophore groups into a single multi-functional molecule [23,24]. In the last decade, coumarin-isoxazole hybrids, among coumarin derivatives, have been synthesized, as they offer diverse biological activities, such as antibacterial [25,26], anticancer [27,28], antiviral, anti-inflammatory, anti-psychotic, antidiabetic [28], antiproliferative [29], antimicrobial [30,31], anticoagulant, and anticholinesterase activities [32]. Hybrids containing coumarin–pyridine scaffold also exhibit a plethora of biological activities, such as anticancer [33,34,35], anti-Alzheimer, antitubercular, antimicrobial, antiviral [36], anti-osteoporotic [37], and antileishmanial activities [38]. Additionally, isoxazole-pyridine hybrids present interesting biological properties, such as anti-acetylcholinesterase [39], anticancer, antioxidant [40], antitubercular [41], and inhibition of human cytochrome P-450 2A6 [42] activities.

An important method for the synthesis of isoxazole derivatives is the Huisgen 1,3-dipolar cycloaddition reaction of nitrile oxides to alkynes, leading to the formation of 3,5-disubstituted isoxazoles [43,44]. The nitrile oxides are formed in situ from the corresponding aldoximes through chlorination and subsequent elimination of HCl by a base or oxidation of aldoxime using an oxidant [40,42]. (Diacetoxyiodo)benzene (PIDA) in room temperature (r.t.) [45], under heating [42], or under microwave irradiation [46] has been utilized for this oxidation. Other analogous reactions use hypochlorous acid at r.t. [39], cerum (IV) ammonium nitrate (CAN) under sonication [41], oxone at r.t. [41], tert-butyl nitrite (TBN) under heating [41], or (bis(trifluoroacetoxy)iodo)benzene (PIFA) under heating [39] as oxidants.

**Scheme 1:**
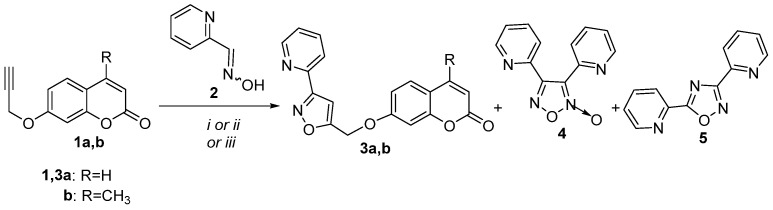
Reaction conditions: (*i*) Method A: propargyl coumarin (1.1 equiv.), PIDA (1.1 equiv.), oxime (1 equiv.) 0.057 M in MeOH; (*ii*) Method B: propargyl coumarin (1.1 equiv.), PIDA (1.1 equiv.), oxime (1 equiv.), EtOH, MW; (*iii*) Method C: propargyl coumarin (1.1 equiv.), TBN (1.1 equiv.), oxime (1 equiv.), MeCN.

**Scheme 2:**
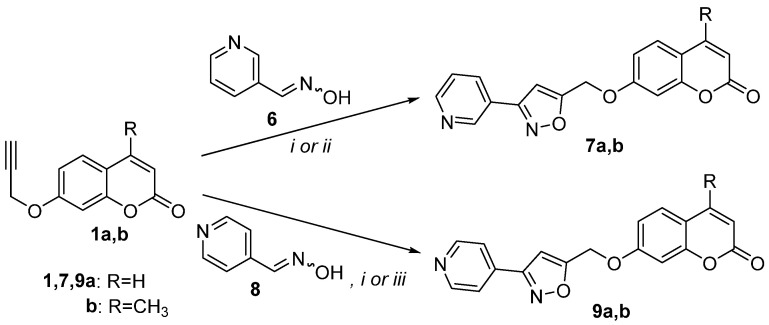
Reaction conditions: (*i*) Method C; (*ii*) Method A with oxime 0.015 M in MeOH; (*iii*) Method D: propargyl coumarin (1.1 equiv.), PIDA (1.1 equiv.), oxime (1 equiv.) 0.015 M in EtOH and TFA (0.5 equiv.).

**Scheme 3:**
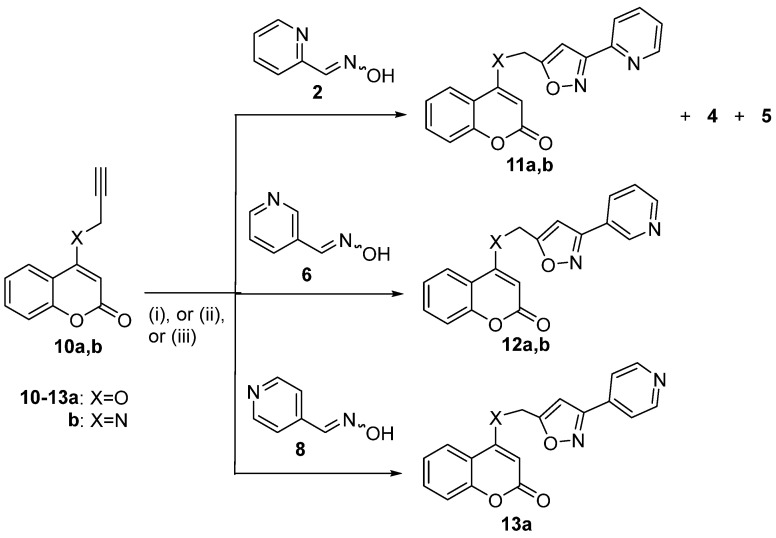
Reaction conditions for (*i*)*,* Method B; (*ii*)*,* Method C; and (*iii*) Method D are referred to in Scheme 1 and Scheme 2 captions.

The available literature suggests that coumarin hybrids containing both coumarin with isoxazole and pyridine moieties do not exist. According to our knowledge, there is little evidence of coumarin hybrids with piperidine, dihydropyridine, or tetrahydropyridine framework. Piperidine hybrids display anti-filovirus [28,47], anti-psychotic [28,48], anti-acetylcholinesterase, and anti-butyrycholinesterase [22,49] activities. 1,4-Dihydropyridine hybrids present antidiabetic activity [28,50]. Previously, 1,2,3,4-tetrahydropyridine-fused coumarin with isooxazoline hybrid has been synthesized via an intramolecular 1,3-dipolar cycloaddition reaction [51]. Herein, in continuation of our ongoing interest in the synthesis and biological evaluation of coumarin hybrids [52,53,54,55], coumarin, isoxazole, and pyridine moieties were selected for hybridization in one molecule to investigate their biological capacity to inhibit lipid peroxidation and particularly enzymes like lipoxygenase (LOX), which are implicated in inflammation. Preliminary in vitro screening tests for LOX inhibition and anti-lipid peroxidation for the new hybrids were performed [56], and a discussion on the structure–activity relationship is presented. The synthesis of the hybrids was achieved by 1,3-dipolar cycloaddition reaction of pyridine aldoximes with propargyloxy- or propargylaminocoumarins. The studied reactions and the isolated products are depicted in Scheme 1, Scheme 2 and Scheme 3.

## 2. Results and Discussion

The 1,3-dipolar cycloaddition reaction of nitrile oxide, generated in situ from picolinaldehyde oxime (**2**) [57], with 1.1 equivalents of 7-propargyloxycoumarin (**1a**) [58], was selected as a model reaction for the investigation of suitable reaction conditions (Scheme 1). At first, PIDA, an efficient and inexpensive oxidizing reagent [45], was utilized for the in situ synthesis of the corresponding pyridine nitrile oxide. The reaction was performed using 1.1 equivalents of PIDA as the oxidant in methanol with a 0.057 M concentration of oxime at room temperature (Method A) to give the new 3,5-disubstituted isoxazole derivative **3a** in 60% yield. The dimerization product of nitrile oxide, furoxan **4** [59] (20%), was also isolated from the reaction mixture (Table 1, entry 1). Almost the same results were observed using a more diluted solution of oxime in a concentration of 0.015 M. HSQC experiments revealed the regiochemistry of **3a**. The 4-H of the isoxazole ring at 7.04 ppm corresponds to 103.1 ppm, as depicted in HSQC, both characteristic of 4-H and 4-C of 3,5-diaryl-substituted isoxazoles [60]. Subsequently, we assessed if the use of microwave (MW) irradiation would affect the reaction’s results. The reaction in ethanol under MW at 120 °C for 1 h (Method B) resulted in isoxazole **3a** in 48% yield, followed by furoxan **4** (16%) and 1,2,4-oxadiazole **5** [61] (9%) (Table 1, entry 2). Compound **5** was possibly formed by the 1,3-dipolar cycloaddition reaction of the nitrile oxide, with the corresponding nitrile obtained by dehydration of aldoxime **2** under the reaction conditions. The dehydration of aldoximes is a convenient route for the synthesis of nitriles under various conditions with a plethora of reagents [62]. A simple procedure, for example, is the transformation of nicotine aldehyde oxime to nicotine nitrile by heating in DMF as solvent at 135 °C [63]. As a third method, we selected TBN, a novel and green oxidizing reagent [41,64], as the oxidant for this reaction with acetonitrile as solvent under reflux for 18 h (Method C). The latter yielded the worst results with isoxazole **3a** (34%) and 1,2,4-oxadiazole **5** (32%) isolated from the reaction mixture (Table 1, entry 3). It was evident that the increase in temperature favors the dehydration of oximes and the formation of 1,2,4-oxadiazole. The similar reactions of oxime **2** with 4-methyl-7-propargyloxycoumarin (**1b**) [58] under Methods A, B, or C resulted in the synthesis and isolation of isoxazole **3b** in 65%, 43%, or 44% yield, respectively, along with furoxan **4** and 1,2,4-oxadiazole **5** (Table 1, entries 4–6). The above results indicate that in the case of picoline aldehyde oximes, Method A gave the better results.

We utilized, then, the nitrile oxide generated from nicotine aldehyde oxime (**6**) [42] in the reactions with 7-propargylocoumarins **1a** and **1b** (Scheme 2). The reactions of **1a** and **1b** under Method Ain a concentration of 0.015 M in MeOH afforded isoxazoles **7a** and **7b** in only 24% and 30% yields, respectively (Table 1, entry 7,9). The best results were under Method Cand led to the synthesis of isoxazoles **7a** and **7b** in 61% and 53% yields, respectively (Table 1, entries 8,10). The yield of these reactions is within the range previously reported for this nitrile oxide [42]. 

The reaction of nitrile oxide prepared from isonicotine aldehyde oxime (**8**) [65] with 7-propargyloxycoumarin (**1a**) was tested next under Method Abut had no results. When trifluoroacetic acid (TFA) (0.5 equiv.) was added with an oxime concentration of 0.015 M in EtOH (Method D), under reflux for 2 days, isoxazole **9a** was isolated from the reaction mixture in 24% yield (Table 1, entry 11). The same reaction using Method C led to isoxazole **9a** in 42% yield (Table 1, entry 12). The analogous reaction of oxime **8** with coumarin **1b** under Method D led to isoxazole **9b** in only 12% yield (Table 1, entry 13), while underMethod C, isoxazole **9b** was isolated in 45% yield (Table 1, entry 14). The above results suggested that Method C is better with the nitrile oxides formed from nicotine aldehyde oxime (**6**) and isonicotine aldehyde oxime (**8**). 

After the examination of 7-propargyloxycoumarins, we tested 1,3-dipolar cycloaddition reactions of 4-propargyloxycoumarin (**10a**) [66] and 4-propargylaminocoumarin (**10b**) [67] with the nitrile oxides formed from pyridine aldoximes (Scheme 3, Table 2). The reaction of **10a** with **2** under Method B at 100 °C afforded isoxazole **11a** (44%) and furoxan **4** (27%) (Table 2, entry 1), whereas under Method C, isoxazole **11a** was synthesized in better yield (62%) accompanied by furoxan **4** (5%) and oxadiazole **5** (11%) (Table 2, entry 2). The similar reaction of **10b** with oxime **2** led to isoxazole **11b** (55%) under Method B (Table 2, entry 3). The analogous reaction of nicotine aldoxime (**6**) with **10a** gave similar results under Method D and Method C, resulting in the isolation of isoxazole **12a** in 30% and 33% yield, respectively (Table 2, entries 4,5). Oxime **6** reacted, also, with 4-propargylaminocoumarin (**10b**) under Method C to give isoxazole **12b** in 56% yield (Table 2, entry 6). Isonicotine aldehyde oxime (**8**) was examined next for the reaction with **10a**. When using PIDA at room temperature (Method A), there were no results. The use of Method D after reflux in ethanol for 2 days led to the isoxazole **13a** in 55% yield (Table 2, entry 7). The same reaction under Method C afforded isoxazole **13a** in 40% yield (Table 2, entry 8). The attempts to trigger a reaction of **10b** with oxime **8** under all the methods examined (Methods A, B, C, and D) were unsuccessful, leaving the 4-propargylaminocoumarin (**10b**) unaffected. The results suggested that Methods B, C, and D produce similar moderate-to-good results for the 4-substituted propargyl coumarin derivatives.

### 2.1. Biology

Isoxazole derivatives of coumarin or pyridine have been proven to exhibit anticancer, antioxidant, and anti-inflammatory properties [27,28,29,40]. Therefore, we decided to evaluate the in vitro behavior of these new coumarin–isoxazole–pyridine hybrids as inhibitors of lipid peroxidation and as anti-inflammatories through the inhibition of soybean lipoxygenase (sLOX). 

We investigated the antioxidant activity of the compounds as inhibitors of the lipid peroxidation of linoleic acid sodium induced by alkyl peroxy free radicals produced by 2,2-azobis(2-amidinopropane) hydrochloride (AAPH). The water-soluble AAPH generates in vitro peroxyl free radicals through spontaneous thermal decomposition. The derived experimental conditions resembled cellular lipid peroxidation due to the activity of the formed radicals. Trolox was used as a reference compound for comparative purposes (Table 3). Among the hybrids, **12b**, which is a 4-substituted amine, presented the highest activity (90.4%), whereas **9b**, **11a**, **9a**, **12a**, **11b**, **and 3a** followed with 86.6, 86, 83, 72, and 66% anti-lipid peroxidation ability, respectively. Additionally, **13a** and **7a** exhibited lower activities, while **3b** was inactive. Lipophilicity does not seem to influence activity. The inhibition of LOX was performed by the UV absorbance-based enzyme assay [68]. The IC_50_ inhibition values in Table 3 showed two potent inhibitors, **13a** and **7a**. Both are ethers from the 7- or 4-position of the coumarin ring and are conjugated to a pyridyl group. The 4-pyridyl derivative **13a** was highly active (IC_50_ = 5 µM). No/low inhibition was shown by the other derivatives. The corresponding amino-substituted derivatives do not possess any activity. It seems that bulk and steric factors are important, and that the stereochemistry of the derivatives influences inhibition more than lipophilicity. Molecular volume and molar refractivity are two physicochemical parameters expressing the overall volume/size and stereochemistry of the molecules, and they play an important role in LOX inhibition. This agrees with previous results presented in a comparative QSAR study on LOX inhibitors [69]. The findings of the publication support that steric factors are important for LOX inhibition since there are steric requirements for the catalytic site of the enzyme.

### 2.2. Biochemistry

Cytotoxic activity against three different cancer cell lines was examined for the more potent isoxazole derivatives **7a**, **9a**, **12b**, and **13a**. HeLa from cervical cancer, HT-29 from colon cancer, and H1437 from lung cancer were utilized to assess the cytotoxic activity of those compounds by using the colorimetric method 3-(4,5-dimethylthiazol-2yl)-2,5 diphenyl tetrazolium bromide (MTT) [70]. The results were expressed as EC_50_ (the concentration that causes 50% loss of cell viability) (Table 4). The results showed that **7a** and **9a** exhibited EC₅₀ values greater than 100 μM in all three cell lines, indicating low cytotoxicity (Table 4, entries 1,2). In contrast, **12b** demonstrated the highest cytotoxicity in HeLa cells (EC₅₀ = 38.1 μM) and a similar effect in H1437 cells (EC₅₀ = 47.3 μM), whereas it was less effective in HT-29 cells (EC₅₀ = 96.5 μM) (Table 4, entry 3). Similarly, **13a** showed moderate cytotoxicity, with EC₅₀ values of 44.2 μM, 65.8 μM, and 74.8 μM in HeLa, HT-29, and H1437 cells, respectively (Table 4, entry 4).

## 3. Materials and Methods

### 3.1. Materials

All the chemicals were purchased from either Sigma-Aldrich Chemie GmbH (Eschenstr. 5, 82024 Taufkirchen (bei Munchen), Germany) or Merck KGaA, (Frankfurter Strasse 250, 64293 Darmstadt, Germany). Melting points were determined with a Kofler hot stage apparatus and are uncorrected. IR spectra were obtained with a PerkinElmer Spectrum BX spectrophotometer as Nujol mulls. NMR spectra were recorded with an Agilent 500/54 (DD2) (500 MHz and 125 MHz for ^1^H and ^13^C, respectively) using TMS as an internal standard. J values are reported in Hz. Mass spectra were determined with an LCMS-2010 EV instrument (Shimadzu, Kyoto, Japan) under electrospray ionization (ESI) conditions. HRMS (ESI-MS) were recorded with a ThermoFisher Scientific (168 Third Avenue, Waltham, MA 02451, USA) model LTQ Orbitrap Discovery MS. Silica gel No. 60 (Merck KGaA, Frankfurter Strasse 250, 64293 Darmstadt, Germany) was used for column chromatography.

### 3.2. Chemistry

#### General Procedure of the 1,3-dipolar Cycloaddition Reactions of Propargyl Coumarins with Pyridine Aldoximes and Synthesis of (3-(pyridin-2-yl)isoxazol-5-yl)methoxy)-2H-chromen-2-one (**3a**)

Method A: A total of 0.1 g (0.5 mmol) of 7-(prop-2-yn-1-yloxy)-2*H*-chromen-2-one (**1a**) was dissolved in methanol (4 mL) under stirring at room temperature. 0.161 g (0.5 mmol) of PIDA was then added. Then, 56 mg (0.455) mmol of picolinaldehyde oxime (**2**) was dissolved in methanol (4 mL), and the solution was added dropwise to the solution of alkyne over a period of 2 h. The reaction was monitored by TLC (hexane:ethyl acetate (EA) (3:1)). 

The reaction was completed 1 h after the addition of oxime. The crude mixture was evaporated, and the residue was separated by column chromatography (hexane:EA (3:1)to EA) to afford 88 mg (60%) of **3a** and 22 mg (20%) of **4**.

Method B: A total of 0.1 g (0.5 mmol) of **1a** was dissolved in ethanol (4 mL) under stirring at room temperature. A total of 0.161 g (0.5 mmol) of PIDA and 56 mg (0.455 mmol) of **2** were added, and the reaction mixture was irradiated under MW irradiation at 120 °C for 1 h. The reaction was monitored by TLC (hexane:EA (3:1)). The mixture was filtered, and the precipitate was washed with hexane (3 × 3 mL) and dried to give 70 mg (48%) of **3a**. The filtrate was evaporated and purified by column chromatography (hexane:EA (3:1))to EA to give 18 mg (16%) of **4** and 10 mg (9%) of **5**.

Method C: In a solution of 0.1 g (0.5 mmol) of **1a** in acetonitrile (8 mL), 56 mg (0.455 mmol) **2**) and 0.006 mL (52 mg, 0.5 mmol) of TBN were added under N_2_ atmosphere at room temperature. The reaction was monitored by TLC (hexane:EA (3:1)). The reaction was refluxed for 18 h. After the completion of the reaction, as indicated by TLC, water (10 mL) was added. The precipitate formed was purified by column chromatography (hexane:EA (1:1) to EA) to give 50 mg (34%) of **3a**. The filtrate was extracted with EA (3 × 15 mL), and the combined organic layers were washed once with brine (20 mL), dried over anhydrous Na_2_SO_4_, filtered, and concentrated. The crude mixture was purified by column chromatography (hexane:EA (3:1) to EA) to afford 33 mg (32%) of **5**.

Method D: A total of 0.1 g (0.5 mmol) of 7-(prop-2-yn-1-yloxy)-2*H*-chromen-2-one (**1a**) was dissolved in methanol (4 mL) under stirring at room temperature. A total of 0.161 g (0.5 mmol) of PIDA was then added, followed by 0.0168 mL (28.5 mg (0.25 mmol)) of TFA. A total of 56 mg (0.455) mmol of isonicotine aldehyde oxime (**6**) was dissolved in methanol (22 mL), and the solution was added dropwise to the solution of alkyne over a period of 2 h. The reaction was monitored by TLC (hexane:EA (3:1)). The reaction was refluxed for 2 days after the addition of oxime. The crude mixture was evaporated, and the residue was separated by column chromatography (hexane:EA (3:1) to EA) to afford 35 mg (24%) of **9a**.

(3-(Pyridin-2-yl)isoxazol-5-yl)methoxy)-2*H*-chromen-2-one (**3a**)

White solid, m.p. 178–179 °C, (hexane/ethyl acetate). ^1^H-NMR (500 MHz, CDCl_3_) δ: 5.30 (s, 2H), 6.29 (d, *J* = 9.5 Hz, 1H) 6.93 (s, 1H), 6.93 (d, *J* = 8.1 Hz, 1H), 7.04 (s, 1H), 7.33–7.38 (m, 1H), 7.42 (d, *J* = 8.2 Hz, 1H), 7.65 (d, *J* = 9.5 Hz, 1H), 7.81 (t, *J* = 7.2 Hz, 1H), 8.09 (d, *J* = 7.8 Hz, 1H), 8.68 (d, *J* = 4.2 Hz, 1H). ^13^C-NMR (126 MHz, CDCl_3_) δ: 61.6, 102.2, 103.1, 112.9, 113.6, 114.1, 121.9, 124.9, 129.2, 137.1, 143.3, 148.1, 149.9, 155.8, 160.8, 161.0, 163.6, 167.4. IR (Nujol): 3086, 1728, 1629 cm^−1^. LC-MS (ESI): (*m*/*z*): 343 [M + Na]^+^. HRMS (ESI): *m*/*z* calcd. for C_18_H_12_N_2_O_4_Na: 321.0870 (M + Na)^+^; found: 321.0898.

4-Methyl-7-((3-(pyridin-4-yl)isoxazol-5-yl)methoxy)-2*H*-chromen-2-one (**3b**).

Mass of 99 mg (65% under Method A), 65 mg (43% under Method B), 67 mg (44% under Method C), white solid, m.p. 173–175 °C, (hexane/ethyl acetate). ^1^H NMR (300 MHz, CDCl_3_) δ: 2.41 (s, 3H), 5.31 (s, 2H), 6.17 (s, 1H), 6.83–7.0 (m, 2H), 7.29 (s, 1H), 7.45– 7.53 (m, 1H), 7.55 (d, *J* = 8.7 Hz, 1H), 7.95 (t, *J* = 7.0 Hz, 1H), 8.20 (d, *J* = 7.9 Hz, 1H), 8.73 (d, *J* = 4.4 Hz, 1H). ^13^C-NMR (126 MHz, DMSO-*d*_6_) δ: 18.2, 60.6, 101.8, 103.4, 11.7, 112.5, 113.9, 121.4, 125.3, 126.7, 137.6, 147.3, 150.0, 153.4, 154.6, 160.0, 160.3, 162.9, 168.1. IR (Nujol): 3085, 1725, 1621 cm^−1^. LCMS (ESI): (*m*/*z*): 357 [M + Na]^+^. HRMS (ESI): *m*/*z* calcd. for C_19_H_14_N_2_O_4_H: 335.1026 (M + H)^+^; found: 335.1054.

7-((3-(Pyridin-3-yl)isoxazol-5-yl)methoxy)-2H-chromen-2-one (**7a**). Mass of 89 mg (61 % under Method C), white solid, m.p. 169–171 °C, (hexane/ethyl acetate). ^1^H NMR (500 MHz, DMSO-*d*_6_) δ: 5.51 (s, 2H), 6.34 (d, *J* = 9.5 Hz, 1H), 7.09 (dd, *J* = 8.6, 2.5 Hz, 1H), 7.20 (d, *J* = 2.4 Hz, 1H), 7.37 (s, 1H), 7.58 (dd, *J* = 8.0, 4.8 Hz, 1H), 7.69 (d, *J* = 8.7 Hz, 1H), 8.02 (d, *J* = 9.5 Hz, 1H), 8.30 (d, *J* = 7.9 Hz, 1H), 8.71 (dd, *J* = 4.8, 1.5 Hz, 1H), 9.10 (d, *J* = 1.6 Hz, 1H). ^13^C NMR (126 MHz, DMSO-*d*_6_) δ: 60.9, 101.8, 102.8, 109.6, 112.9, 113.1, 124.3, 124.4, 129.7, 134.4, 144.3, 147.5, 151.2, 155.2, 159.9, 160.2, 160.5, 168.3. IR (Nujol): 3084, 1710, 1625 cm^−1^. LCMS (ESI): (*m*/*z*): 321 [M + H]^+^, 343 [M + Na]^+^. HRMS (ESI): *m*/*z* calcd. for C_18_H_12_N_2_O_4_H: 321.0870 (M + H)^+^; found: 321.0866.

4-Methyl-7-((3-(pyridin-3-yl)isoxazol-5-yl)methoxy)-2*H*-chromen-2-one (**7b**). Mass of 81 mg (53 % under Method C), white solid, m.p. 150–152 °C, (hexane/ethyl acetate). ^1^H NMR (500 MHz, DMSO-*d*_6_) δ: 2.40 (s, 3H), 5.29 (s, 2H), 6.17 (s, 1H), 6.75 (s, 1H), 6.92 (d, *J*=2.0 Hz 1H), 6.95 (dd, *J* = 8.8, 2.0 Hz, 1H), 7.42 (dd, *J* = 7.4, 5.0 Hz, 1H), 7.55 (d, *J* = 8.7 Hz, 1H), 8.16 (d, *J* = 7.8 Hz, 1H), 8.70 (d, *J* = 3.3 Hz, 1H), 9.02 (s, 1H). ^13^C NMR (126 MHz, CDCl_3_) δ: 18.8, 61.5, 101.8, 102.1, 112.5, 112.9, 114.8, 124.0, 124.9, 126.1, 134.3, 148.0, 151.3, 152.4, 155.2, 160.2, 160.5, 161.1, 168.1. IR (Nujol): 3090, 1710, 1620 cm^−1^. LCMS (ESI): (*m*/*z*): 357 [M + Na]^+^. HRMS (ESI): *m*/*z* calcd. for C_19_H_14_N_2_O_4_H: 335.1026 (M + H)^+^; found: 335.1018.

7-((3-(Pyridin-4-yl)isoxazol-5-yl)methoxy)-2*H*-chromen-2-one (**9a**). Mass of 35 mg (24 % under Method D), 61 mg (42% under Method C), white solid, m.p. 181–183 °C, (hexane/ethyl acetate). ^1^H NMR (500 MHz, DMSO-*d*_6_) δ: 5.38 (s, 2H), 6.20 (d, *J* = 9.5 Hz, 1H), 6.95 (dd, *J* = 8.6, 2.4 Hz, 1H), 7.00 (d, *J* = 2.4 Hz, 1H), 7.18 (s, 1H), 7.50(dd, *J* = 8.6, 3.5 Hz, 1H), 7.78 (dd, *J* = 9.2, 4.6 Hz, 1H), 7.96 (d, *J* = 4.1 Hz, 2H), 8.77 (d, *J* = 4.1 Hz, 2H). ^13^C NMR (126 MHz, CDCl_3_/DMSO-*d*_6_) δ: 60.6, 101.4, 102.4, 106.3, 112.3, 112.88, 112.94, 121.6, 129.0, 143.3, 147.8, 155.1, 159.5, 159.9, 160.1. IR (Nujol): 3090, 1715, 1628 cm^−1^. LCMS (ESI): (*m*/*z*): 321[M + H]^+^. HRMS (ESI): *m*/*z* calcd. for C_18_H_12_N_2_O_4_H: 321.0870 (M + H)^+^; found: 321.0857. *m*/*z* calcd. for C_18_H_12_N_2_O_4_Na: 343.0689 (M + Na)^+^; found: 343.0684.

4-Methyl-7-((3-(pyridin-4-yl)isoxazol-5-yl)methoxy)-2*H*-chromen-2-one (**9b**). Mass of 68 mg (45% under Method C), white solid, m.p. 180–181 °C, (hexane/ethyl acetate). ^1^H NMR (500 MHz, DMSO-*d*_6_) δ: 2.41 (s, 3H), 5.53 (s, 2H), 6.25 (s, 1H), 7.10 (d, *J* = 8.7 Hz, 1H), 7.18 (s, 1H), 7.38 (s, 1H), 7.74 (d, *J* = 8.8 Hz, 1H), 7.87 (d, *J* = 5.1 Hz, 2H), 8.75 (d, *J* = 5.0 Hz, 2H). ^13^C NMR (126 MHz, DMSO-*d*_6_) δ: 18.1, 60.8, 101.8, 102.9, 111.7, 112.5, 113.9, 120.9, 126.7, 128.2, 128.9, 135.4, 150.7, 153.3, 154.6, 160.0, 160.3, 160.5, 168.8. IR (Nujol): 3086, 1718, 1630 cm^−1^. LCMS (ESI): (*m*/*z*): 357 [M+Na]^+^. HRMS (ESI): *m*/*z* calcd. for C_19_H_14_N_2_O_4_H: 335.1026 (M + H)^+^; found: 335.1015.

4-((3-(Pyridin-2-yl)isoxazol-5-yl)methoxy)-2*H*-chromen-2-one (**11a**). Mass of 64 mg (44% under Method A), 90 mg (62% under Method C), white solid, m.p. 164–165 °C, (hexane/ethyl acetate). ^1^H NMR (500 MHz, DMSO-*d*_6_) δ: 5.67 (s, 2H), 6.20 (s, 1H), 7.34 (s, 1H), 7.38 (t, *J* = 7.6 Hz, 1H), 7.43 (d, *J* = 8.3 Hz, 1H), 7.54–7.59 (m, 1H), 7.69 (t, *J* = 7.8 Hz, 1H), 7.86 (d, *J* = 7.9 Hz, 1H), 7.99 (td, *J* = 7.7, 1.1 Hz, 1H), 8.07 (d, *J* = 7.8 Hz, 1H), 8.74 (d, *J* = 4.2 Hz, 1H). ^13^C NMR (126 MHz, DMSO-*d*_6_) δ: 61.6, 91.7, 103.7, 114.8, 116.5, 121.5, 122.9, 124.4, 125.3, 133.0, 137.6, 147.2, 150.0, 152.8, 161.4, 163.0, 164.0, 166.9. IR (Nujol): 3070, 1725, 1620 cm^−1^. LCMS (ESI): (*m*/*z*): 343 [M + Na]^+^. HRMS (ESI): *m*/*z* calcd. for C_18_H_12_N_2_O_4_H: 321.0870 (M + H)^+^; found: 321.0883.

4-(((3-(Pyridin-2-yl)isoxazol-5-yl)methyl)amino)-2*H*-chromen-2-one (**11b**). Mass of 80 mg (55% under Method B), whitish solid, m.p. 196–198 °C, (hexane/ethyl acetate). ^1^H NMR (500 MHz, CDCl_3_) δ: 5.38 (s, 2H), 5.83 (s, 1H), 7.13 (s, 1H), 7.28 (t, *J* = 7.6 Hz, 1H), 7.33 (d, *J* = 8.2 Hz, 1H), 7.38 (dd, *J* = 6.9, 5.3 Hz, 1H), 7.57 (d, *J* = 7.7 Hz, 1H), 7.72–7.90 (m, 1H), 8.11 (d, *J* = 7.8 Hz, 1H), 8.69 (d, *J* = 4.4 Hz, 1H). ^13^C NMR (126 MHz, CDCl_3_) δ: 61.7, 91.6, 103.7, 115.3, 117.0, 121.9, 123.2, 124.3, 125.0, 133.0, 137.2, 147.9, 149.9, 153.5, 162.4, 163.7, 164.7, 165.7; IR (Nujol): 3288, 3085, 1711, 1629 cm^−1^. LCMS (ESI): (*m*/*z*): 343 [M + H + Na]^+^. HRMS (ESI): *m*/*z* calcd. for C_18_H_13_N_3_O_3_H: 320,103 (M + H)^+^; found: 320.1057. *m*/*z* calcd. for C_18_H_13_N_3_O_3_Na: 342.0849 (M+Na)^+^; found: 342.0868.

4-((3-(Pyridin-3-yl)isoxazol-5-yl)methoxy)-2*H*-chromen-2-one (**12a**). Mass of 44 mg (30% under Method D), 48 mg (33% under Method C), white solid, m.p. 189–191 °C, (hexane/ethyl acetate). ^1^H NMR (500 MHz, CDCl_3_) δ: 5.38 (s, 2H), 5.84 (s, 1H), 6.85 (s, 1H), 7.31 (t, *J* = 7.5 Hz, 1H), 7.36 (d, *J* = 8.3 Hz, 1H), 7.46 (dd, *J* = 6.9, 4.8 Hz, 1H), 7.59 (t, *J* = 7.6 Hz, 1H), 7.86 (d, *J* = 7.9 Hz, 1H), 8.20 (d, *J* = 7.7 Hz, 1H), 8.73 (d, *J* = 3.3 Hz, 1H), 9.05 (s, 1H). ^13^C NMR (126 MHz, CDCl_3_/ DMSO-*d*_6_) δ: 61.2, 91.3, 102.7, 114.7, 116.0, 123.7, 127.7, 128.5, 132.3, 145.4, 148.6, 152.6, 157.2, 159.0, 161.3, 163.8, 166.5. IR (Nujol): 3080, 1712, 1630 cm^−1^. LCMS (ESI): (*m*/*z*): 343 [M + Na]^+^. HRMS (ESI): *m*/*z* calcd. for C_18_H_12_N_2_O_4_H: 321.0870 (M + H)^+^; found: 321.0852.

4-(((3-(Pyridin-3-yl)isoxazol-5-yl)methyl)amino)-2*H*-chromen-2-one (**12b**). Mass of 9 mg (6% under Method D), 84 mg (56% under Method C), white solid, m.p. 197–199 °C, (hexane/ethyl acetate); ^1^H NMR (500 MHz, DMSO-*d*_6_) δ: 4.79 (s, 2H), 5.31 (s, 1H), 7.17 (s, 1 H), 7.34 (d, *J =* 8.4 Hz, 1H), 7.37 (t, *J* = 7.3 Hz, 1H), 7.53 (dd, *J* = 7.9, 4.8 Hz, 1H), 7.63 (t, *J* = 8.3 Hz, 1H), 8.10 (d, *J* = 8.0 Hz, 1H), 8.2–8.30 (m, 1H), 8.45 (s, 1H), 8.68 (d, *J* = 3.5 Hz, 1H), 9.07 (d, *J* = 1.3 Hz, 1H), ^13^C NMR (126 MHz, DMSO-*d*_6_) δ: 38.1, 83.1, 100.9, 114.4, 117.0, 122.6, 123.6, 124.2, 124.5, 132.2, 134.1, 147.5, 151.2, 153.09, 153.12, 159.8, 161.4, 170.2; IR (Nujol): 3320, 3085, 1710, 1625. cm^−1^. LCMS (ESI): (*m*/*z*): 342 [M + Na]^+^. HRMS (ESI): *m*/*z* calcd. for C_18_H_13_N_3_O_3_H: 320.1030 (M + H)^+^; found: 320.1030.

4-((3-(Pyridin-4-yl)isoxazol-5-yl)methoxy)-2*H*-chromen-2-one (**13a**). Mass of 80 mg (55% under Method D), 58 mg (40% under Method C), m.p. 167–169 °C, (hexane/ethyl acetate). ^1^H NMR (500 MHz, DMSO-*d*_6_) δ: 5.67 (s, 1H), 6.17 (s, 1H), 7.39 (t, *J* = 7.6 Hz, 1H), 7.44 (d, *J* = 8.3 Hz, 1H), 7.52 (s, 1H), 7.68 (d, *J* = 7.7 Hz, 1H), 7.83–7.92 (m, 1H), 8.77 (d, *J* = 4.8 Hz, 1H). ^13^C NMR (126 MHz, DMSO-*d*_6_) δ: 61.9, 91.8, 103.1, 114.8, 116.5, 121.0, 123.0, 124.4, 133.0, 135.3, 150.7, 152.8, 160.7, 161.4, 163.9, 167.7. IR (Nujol): 3090, 1704, 1640 cm^−1^. LCMS (ESI): (*m*/*z*): 321 [M + H]^+^. HRMS (ESI): *m*/*z* calcd. for C_18_H_12_N_2_O_4_H: 321.0870 (M + H)^+^; found: 321.0871.

### 3.3. Biological Experiments

The in vitro assays were performed at a concentration of 100 µM (a 10 mM stock solution in DMSO was used, from which several dilutions were made for the determination of IC_50_ values) at least in triplicate, and the standard deviation of absorbance was less than 10% of the mean. The compounds were diluted in 0.1% DMSO under sonification in an appropriate buffer in several dilutions (Table 2). Statistical comparisons were made using the Student’s *t*-test. A statistically significant difference was defined as *p* < 0.05.

#### 3.3.1. Inhibition of Linoleic Acid Peroxidation

The in vitro study was evaluated as reported previously by our group [40]. A total of 10 microliters of the 16 mM sodium linoleate solution were added to the UV cuvette containing 0.93 mL of a 0.05 M phosphate buffer, pH 7.4, pre-thermostated at 37 °C. The oxidation reaction was initiated at 37 °C under air by the addition of 50 μL of a 40 mM AAPH solution, which was used as a free radical initiator. Oxidation was carried out in the presence of the samples (10 μL from the stock solution of each compound) in the assay without antioxidants and monitored at 234 nm. Lipid oxidation was recorded in the presence of the same level of DMSO and served as a negative control. Trolox was used as the appropriate reference compound (Table 2).

#### 3.3.2. Soybean Lipoxygenase Inhibition Study

The in vitro study was evaluated as reported previously by our group [59]. The tested compounds were incubated in a tris buffer pH 9, at room temperature, with sodium linoleate (0.1 mM) and 0.2 mL of enzyme solution (1/9 × 10^−4^ *w*/*v* in saline, 1000 U/mL) for 5 min, and after that, the inhibition was measured. The method was based on the conversion of sodium linoleate to 13-hydroperoxylinoleic acid at 234 nm by the appearance of the conjugated diene. Nor-dihydroguaeretic acid NDGA (IC_50_ = 0.45 μM) was used as a reference compound. Different concentrations were used to determine the IC_50_ values. A blank determination was used first to serve as a negative control. The results are given in Table 2.

### 3.4. Biochemical Experiments

#### 3.4.1. Cell Culture

HeLa (cervical cancer), HT-29 (colorectal cancer), and H1437 (lang adenocarcinoma) cell lines were obtained from American Type Culture Collection (ATCC) and cultured in Dulbecco’s Modified Eagle Medium (DMEM). All media were supplemented with 10% fetal bovine serum (FBS) and antibiotic/antimytotic as monolayers at 37 °C in a 5% CO_2_ incubator in a humidified atmosphere. 

#### 3.4.2. Cytotoxicity Evaluation

Cell viability was assessed using the MTT assay. Briefly, cells were seeded in 96-well plates at a density of 3 × 10^3^ cells per well for HeLa and H1437 and 4 × 10^3^ cells per well for HT-29 in 100 μL of complete medium. After 24 h of incubation for cell attachment, the cells were treated with varying concentrations of test compounds (ranging from 10 to 100 μM) for 48 h. Following the treatment period, MTT colorimetric assay was performed as described before [62]. Cell viability was calculated as a percentage of untreated control. The half-maximal effective concentration (EC_50_) values are defined as the concentration that causes a 50% reduction in cell viability relative to controls. All experiments were performed in triplicate and repeated independently at least three times. The ±SE values were calculated. 

## 4. Conclusions

Coumarin–isoxazole hybrids connected to a pyridine framework have been synthesized in moderate-to-good yields by the 1,3-dipolar cycloaddition reaction of nitrile oxides, derived in situ from pyridine aldehyde oximes under oxidation by PIDA or TBN, with 4- or 7-propargyloxycoumarins or 4-propargylaminocoumarins. The 4-pyridyl hybrid **13a** has been found to be a potent inhibitor of LOX with IC_50_ = 5 µM, followed by the 3-pyridyl hybrid **7a** with IC_50_ = 10 µM. Since lipoxygenases and their catalysis products are related to carcinogenic processes such as cell proliferation, differentiation, and apoptosis, the potent hybrid **13a** will be used as a lead compound for further theoretical structural modifications, and in vitro assays. Compounds **12b** and **13a** presented moderate to minimal impact on HeLa, HT-29, and H1437 cancer cells. However, hybrid **12b** presented a combination of high antilipid peroxidation and a moderate impact on HeLa cells and is considered to lead to the design of new hybrids with a higher impact on HeLa. Further work is underway to delineate the role of these hybrids in inflammation. 

## Figures and Tables

**Table 1:** 1,3-Dipolar cycloaddition reactions of pyridine nitrile oxides with 7-propargyloxycoumarins.

Entry	Oxime	7-Propagyl Oxycoumarin	Method ^1^	Temperature	Time	Products (% Yield)
1	**2**	**1a**	A	r.t.	1 h	**3a** (60), **4** (20)
2	**2**	**1a**	B	120 °C	1 h	**3a** (48), **4** (16), **5** (9)
3	**2**	**1a**	C	Reflux	18 h	**3a** (34), **5** (32)
4	**2**	**1b**	A	r.t.	15 h	**3b** (65), **4** (17)
5	**2**	**1b**	B	120 °C	1 h	**3b** (43), **4** (17), **5** (11)
6	**2**	**1b**	C	Reflux	18 h	**3b** (44), **4** (15), **5** (13)
7	**6**	**1a**	A ^2^	r.t.	18 h	**7a** (24)
8	**6**	**1a**	C	Reflux	18 h	**7a** (61)
9	**6**	**1b**	A ^2^	r.t.	18 h	**7b** (30)
10	**6**	**1b**	C	Reflux	18 h	**7b** (53)
11	**8**	**1a**	D	Reflux	2 d	**9a** (24)
12	**8**	**1a**	C	Reflux	18 h	**9a** (42)
13	**8**	**1b**	D	Reflux	2 d	**9b** (12)
14	**8**	**1b**	C	Reflux	18 h	**9b** (45)

^1^ Methods A, B, C, and D are referred to in Scheme 1 and Scheme 2 captions. ^2^ Concentration of 0.015 M in MeOH.

**Table 2:** 1,3-Dipolar cycloaddition reactions of pyridine nitrile oxides with 4-propargylcoumarins.

Entry	Oxime	4-Propargylcoumarin	Method ^1^	Temperature	Time	Products (% Yield)
1	**2**	**10a**	B	100 °C	1 h	**11a** (44), **4** (27)
2	**2**	**10a**	C	Reflux	18 h	**11a** (62), **4** (5), **5** (11)
3	**2**	**10b**	B	100 °C	2 h	**11b** (55)
4	**6**	**10a**	D	Reflux	2 d	**12a** (30)
5	**6**	**10a**	C	Reflux	18 h	**12a** (33)
6	**6**	**10b**	C	Reflux	18 h	**12b** (56)
7	**8**	**10a**	D	Reflux	2 d	**13a** (55)
8	**8**	**10a**	C	Reflux	18 h	**13a** (40)

^1^ Methods B, C, and D are referred to in Scheme 1 and Scheme 2 captions.

**Table 3:** In vitro activities of compounds. Inhibition of soybean lipoxygenase (LOX). (%)/IC_50_ µM. % Inhibition of lipid peroxidation (ILP) at 100 µM.

Entry	Compounds ^1^	Clog *P* ^2^	LOX(%)/ IC_50_ µM	ILP (%)
1	**3a**	2.27	no	66
2	**3b**	2.77	no	0.6
3	**7a**	2.27	10 µM	42
4	**7b**	2.27	no	2
5	**9a**	2.77	38	83.6
6	**9b**	2.77	no	86.6
7	**11a**	2.27	no	86
8	**11b**	1.95	no	66
9	**12a**	2.01	10	72
10	**12b**	1.95	18	90.4
11	**13a**	2.01	5 µM	44.6
12	**NDGA**		0.45 μΜ	
13	**Trolox**			93

^1^ Compounds tested at 100 µM. Values are means ± SD of three or four different. determinations. Means within each column differ significantly (*p* < 0.05); no result was given under the reported experimental conditions. ^2^ Biobyte BioByte Corporation, C-QSAR database, 201 W Fourth Str., Suite # 204, Claremont. CA 91711-4707, USA.

**Table 4:** Half maximal effective concentration (EC_50_ Values) of **7a**, **9a**, **12b**, and **13a** in HeLa, HT-29, and H1437 cancer cell lines. Results are presented as a means ±SE of three independent experiments.

Entry	Compound	HeLa, EC_50_ (μM)	HT-29, EC_50_ (μM)	H1437, EC_50_ (μM)
1	**7a**	>100	>100	>100
2	**9a**	>100	>100	>100
3	**12b**	38.1 ± 2.1	96.5 ± 6.6	47.3 ± 3.1
4	**13a**	44.2 ± 1.9	65.8 ± 5.4	74.8 ± 4.4

## Data Availability

The original contributions presented in this study are included in the article/Supplementary Materials. Further inquiries can be directed to the corresponding author(s).

## Supplementary Materials

The following supporting information can be downloaded at: https://www.mdpi.com/article/10.3390/molecules30071592/s1 ^1^H-NMR and ^13^C-NMR spectra of the compounds and the possible mechanistic schemes for dehydration of picoline aldehyde oxime (**2**) with PIDA or TBN under heating.

## Abbreviations

The following abbreviations are used in this manuscript:

DMSO	Dimethyl Sulfoxide
SD	Standard Deviation
TLC	Thin Layer Chromatography
