# Supplementary material for: Pharmacochemical Studies of Synthesized Coumarin–Isoxazole–Pyridine Hybrids
Source: Molecules. 2025 Apr 2;30(7):1592. doi: 10.3390/molecules30071592 (PMC11990191; doi:10.3390/molecules30071592)

Pharmacochemical Studies of Synthesized Coumarin–Isoxazole–Pyridine Hybrids

Matina D. Douka [1], Ioanna M. Sigala [2], Catherine Gabriel [3,4], Eleni Nikolakaki [2], Dimitra J. Hadjipavlou-Litina [5] and Konstantinos E. Litinas [1,*]

[1] Laboratory of Organic Chemistry, Department of Chemistry, Aristotle University of Thessaloniki, 54124 Thessaloniki, Greece; doukamatina@chem.auth.gr
[2] Laboratory of Biochemistry, Department of Chemistry, Aristotle University of Thessaloniki, 54124 Thessaloniki, Greece; isigala@chem.auth.gr (I.M.S.); nikol@chem.auth.gr (E.N.)
[3] HERACLES Research Centre on the Exposome and Health, Center for Interdisciplinary Research and Innovation, Balkan Center, Thermi, 57001 Thessaloniki, Greece; katerinagabriel@cheng.auth.gr
[4] Laboratory of Environmental Engineering, Department of Chemical Engineering, Aristotle University of Thessaloniki, 54124 Thessaloniki, Greece
[5] Laboratory of Pharmaceutical Chemistry, School of Pharmacy, Faculty of Health Sciences, Aristotle University of Thessaloniki, 54124 Thessaloniki, Greece; hadjipav@pharm.auth.gr
* Correspondence: klitinas@chem.auth.gr; Tel.: +30-2310997864

^{1}H-NMR and ^{13}C-NMR Spectra for the compounds:

(3-(Pyridin-2-yl)isoxazol-5-yl)methoxy)-2*H*-chromen-2-one (**3a**)……………………………………3
4-Methyl-7-((3-(pyridin-4-yl)isoxazol-5-yl)methoxy)-2H-chromen-2-one (**3b**)………………..5
7-((3-(Pyridin-3-yl)isoxazol-5-yl)methoxy)-2H-chromen-2-one (**7a**)……………………………6
4-Methyl-7-((3-(pyridin-3-yl)isoxazol-5-yl)methoxy)-2H-chromen-2-one (**7b**)………………..7
7-((3-(Pyridin-4-yl)isoxazol-5-yl)methoxy)-2H-chromen-2-one (**9a**)……………………………8
4-Methyl-7-((3-(pyridin-4-yl)isoxazol-5-yl)methoxy)-2H-chromen-2-one (**9b**)………………..9
4-((3-(Pyridin-2-yl)isoxazol-5-yl)methoxy)-2H-chromen-2-one (**11a**)……………………….....10
4-(((3-(Pyridin-2-yl)isoxazol-5-yl)methyl)amino)-2H-chromen-2-one (**11b**)………………….11
4-((3-(Pyridin-3-yl)isoxazol-5-yl)methoxy)-2H-chromen-2-one (**12a**)…………………………12
4-(((3-(Pyridin-3-yl)isoxazol-5-yl)methyl)amino)-2H-chromen-2-one (**12b**)………………….13
4-((3-(Pyridin-4-yl)isoxazol-5-yl)methoxy)-2H-chromen-2-one (**13a**)…………………………14
HRMS of **3a**……………………………………………………………………………….15
HRMS of **3b**……………………………………………………………………………….15
HRMS of **7a**……………………………………………………………………………….16
HRMS of **7b**……………………………………………………………………………….16
HRMS of **9a**……………………………………………………………………………….17
HRMS of **9b**……………………………………………………………………………….17
HRMS of **11a**………………………………………………………………………………18
HRMS of **11b**………………………………………………………………………………18
HRMS of **12a**………………………………………………………………………………19
HRMS of **12b**………………………………………………………………………………19
HRMS of **13a**………………………………………………………………………………19
Possible mechanistic Schemes for the dehydration of picoline aldehyde oxime (**2**) in the presence of PIDA or TBN under heating…………………………………………………………20

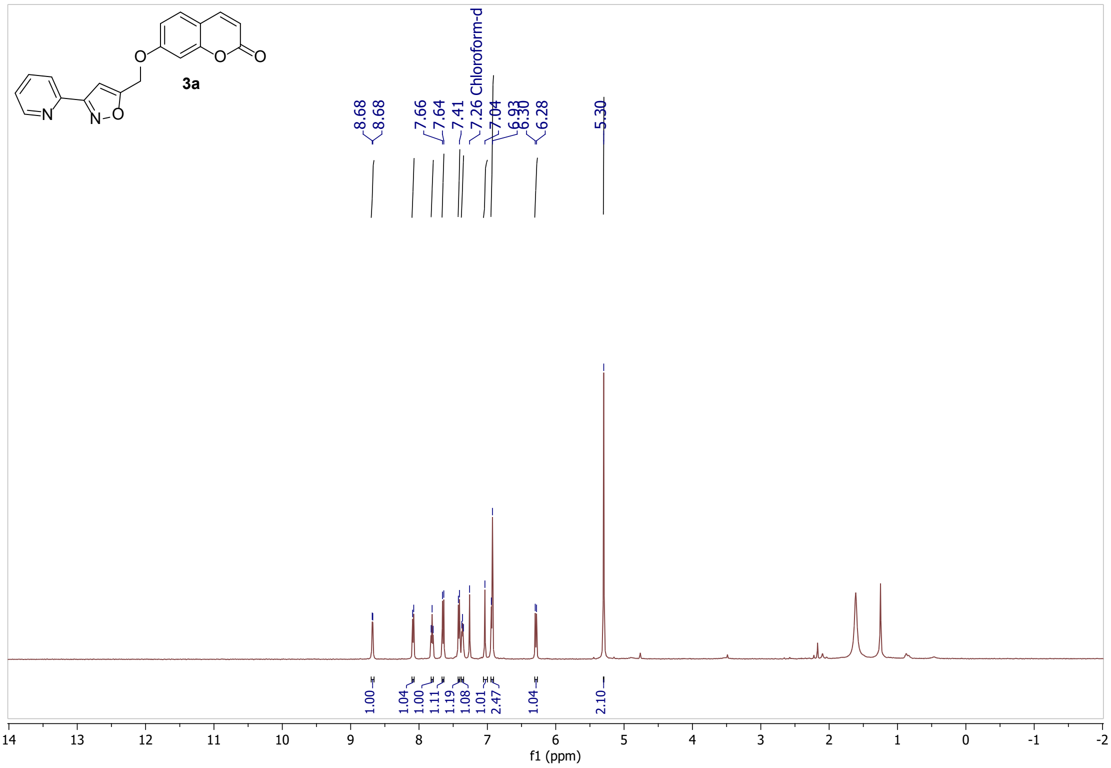

Figure S1. ^{1}H-NMR spectrum of **3a** (500 MHz, $CDCl_3$).

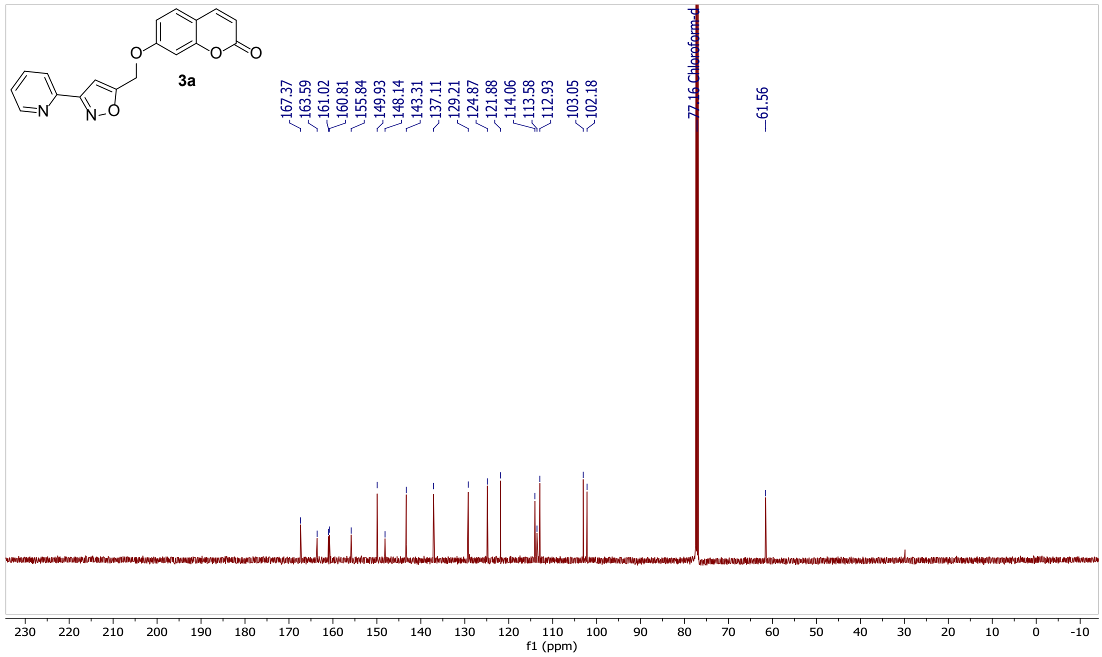

Figure S2. ^{13}C-NMR spectrum of **3a** (500 MHz, $CDCl_3$).

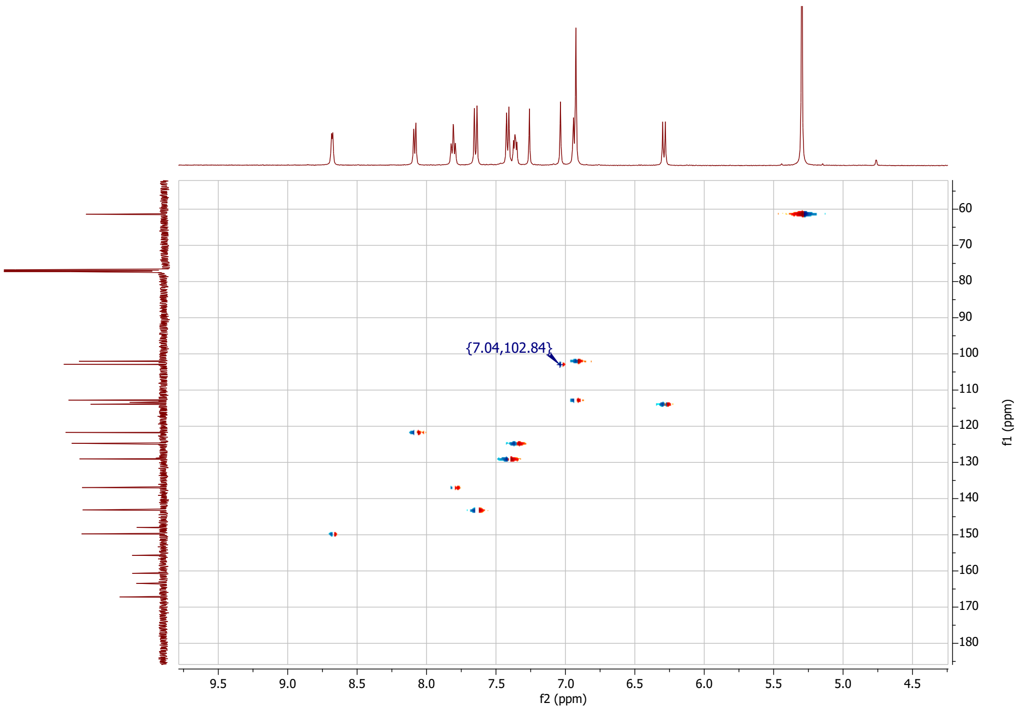

Figure S3. HSQC spectrum of **3a** (500 MHz, $CDCl_3$).

\

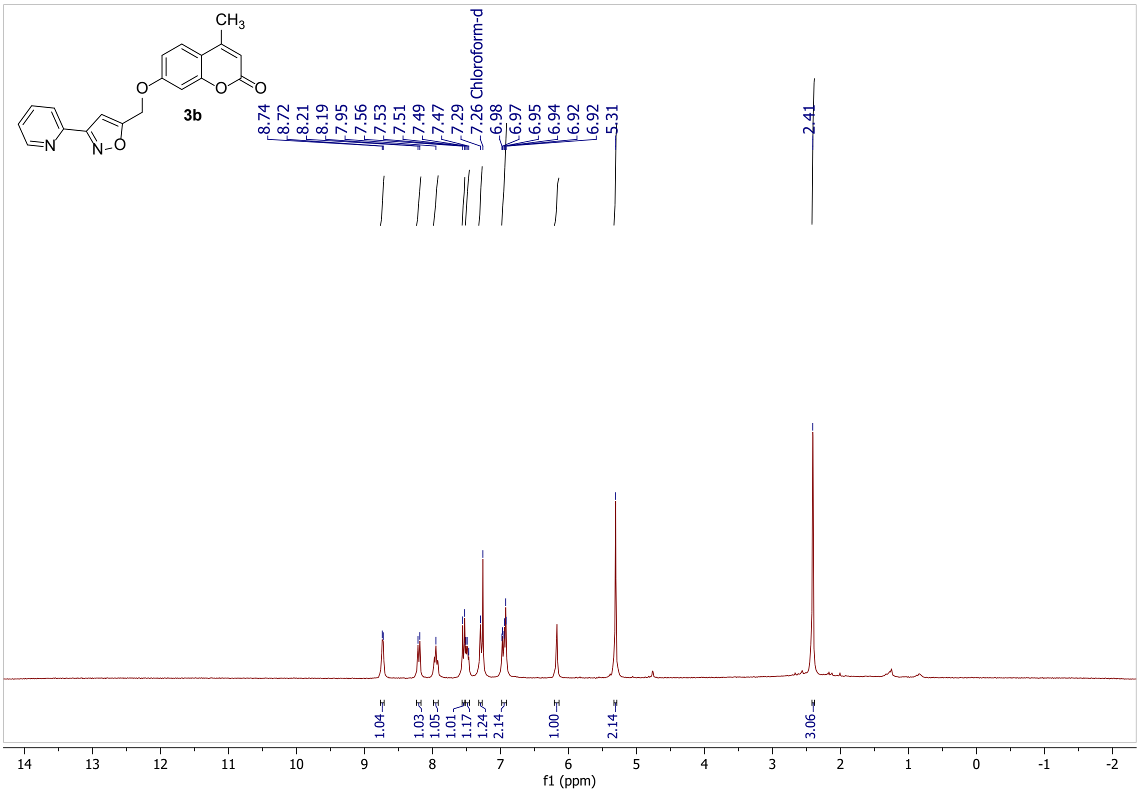

Figure S4. ^{1}H-NMR spectrum of **3b** (500 MHz, $CDCl_3$).

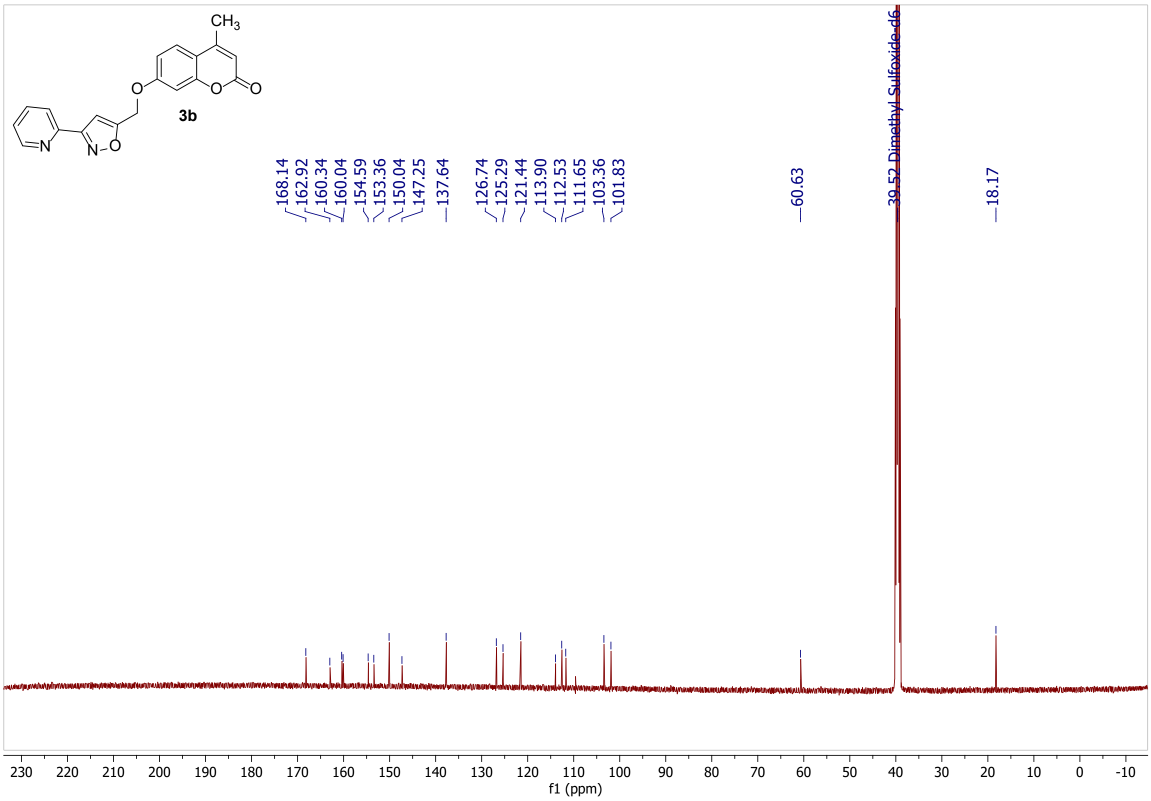

Figure S5. ^{13}C-NMR spectrum of **3b** (500 MHz, DMSO-d_6)

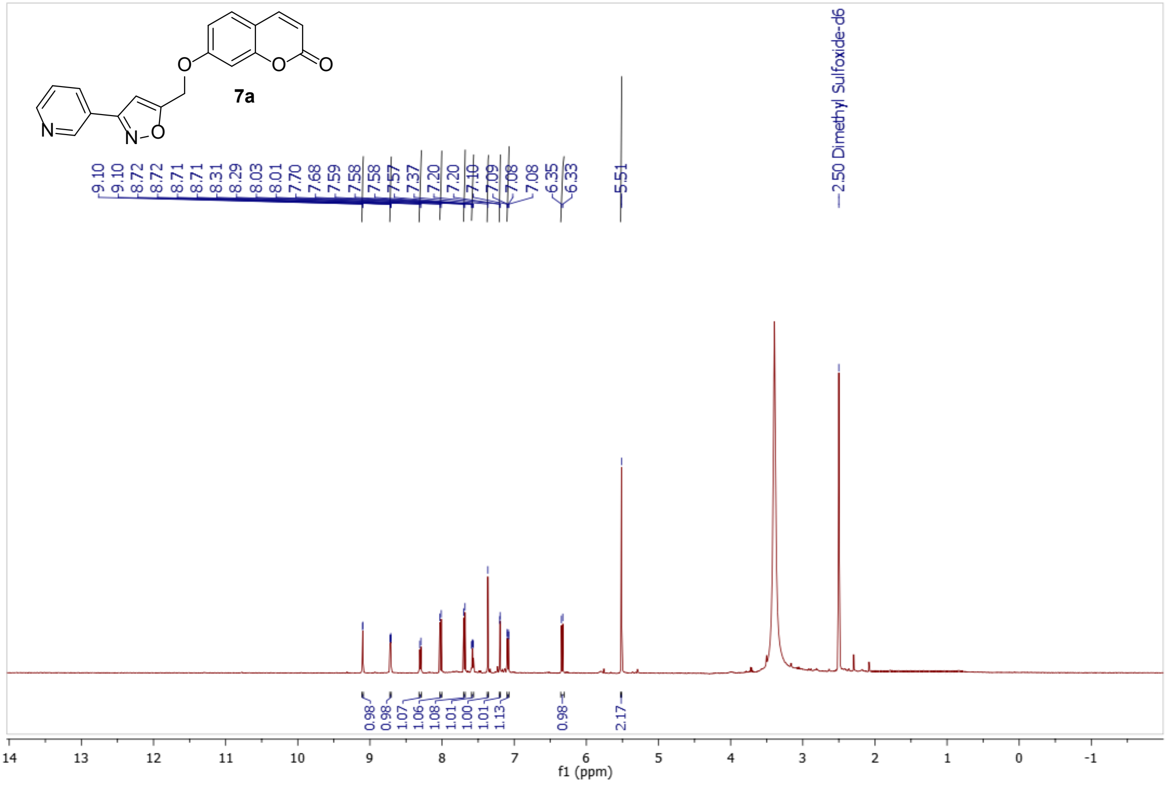

Figure S6. ^{1}H-NMR spectrum of **7a** (500 MHz, DMSO-d_6).

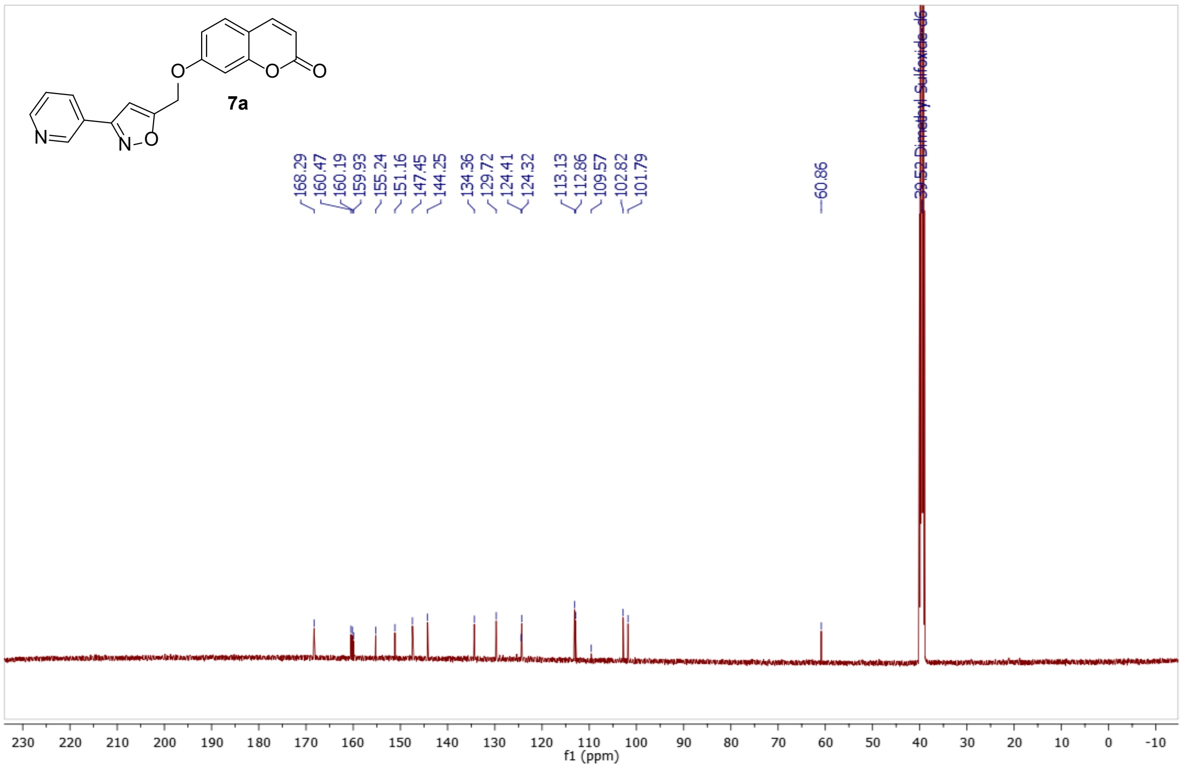

Figure S7. ^{13}C-NMR spectrum of **7a** (500 MHz, DMSO-d_6).

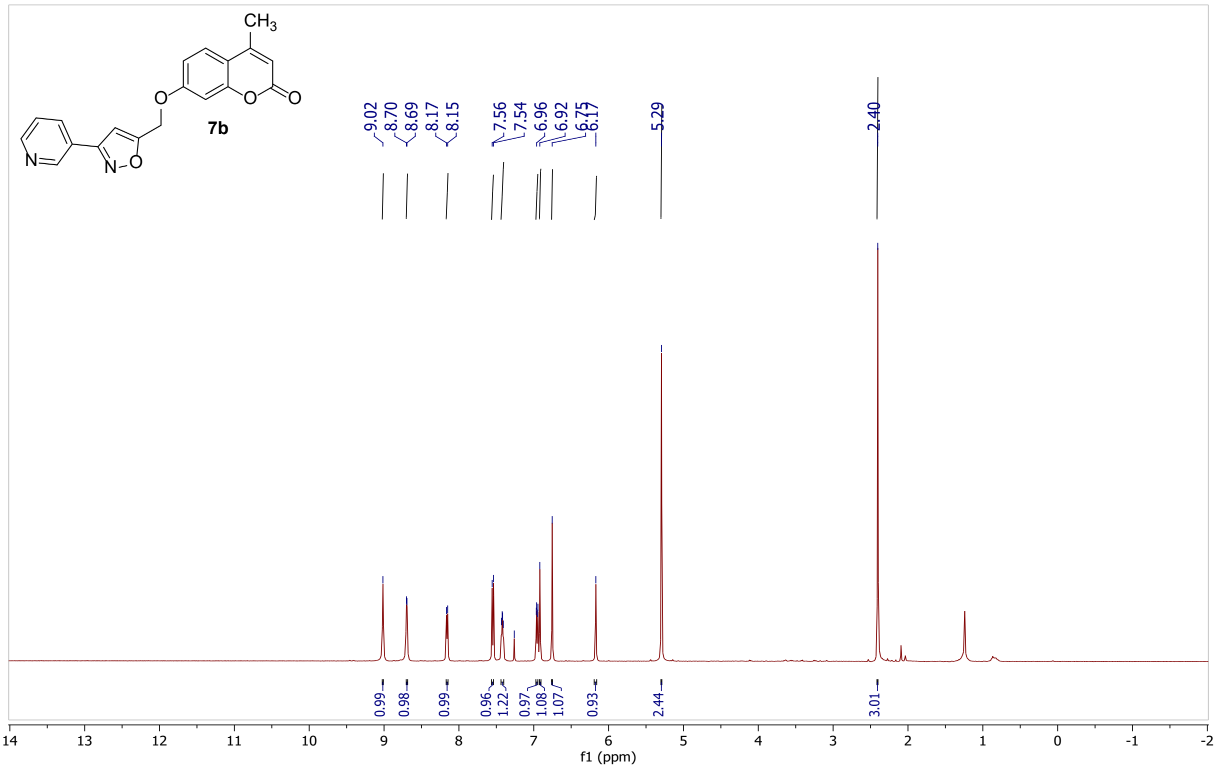

Figure S8. ^{1}H-NMR spectrum of **7b** (500 MHz, $CDCl_3$).

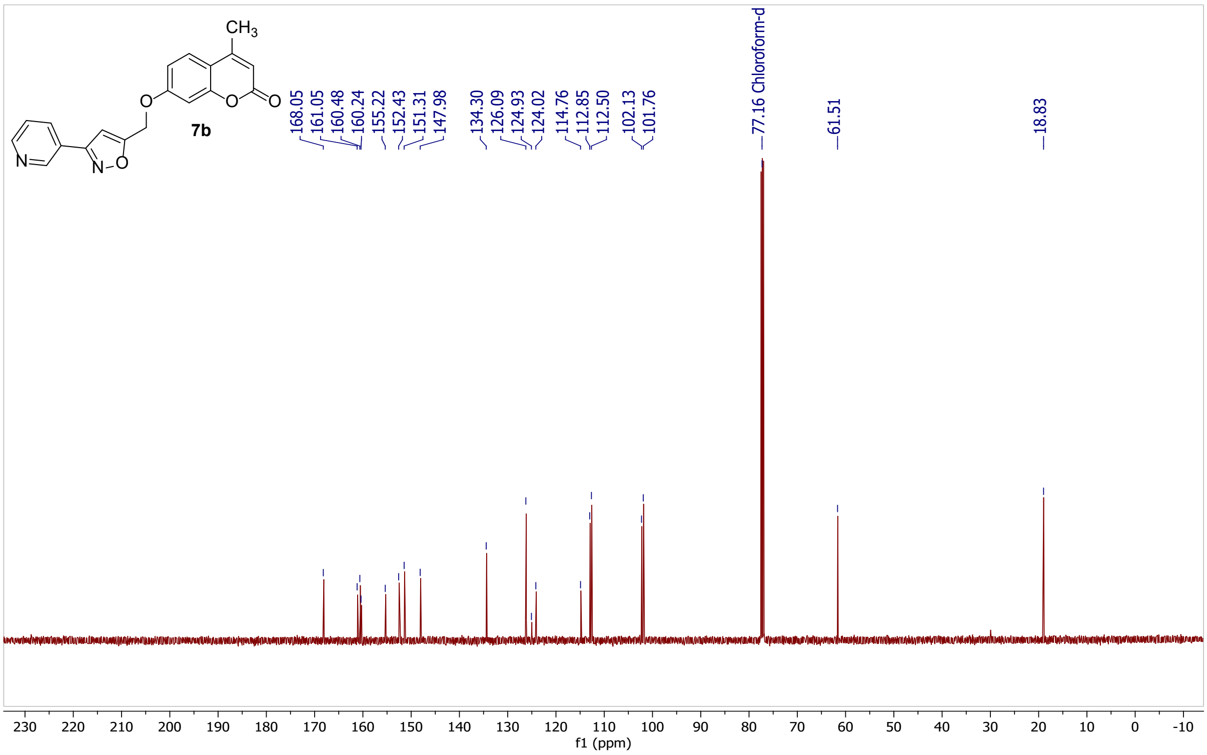

Figure S9. ^{13}C-NMR spectrum of **7b** (500 MHz, $CDCl_3$).

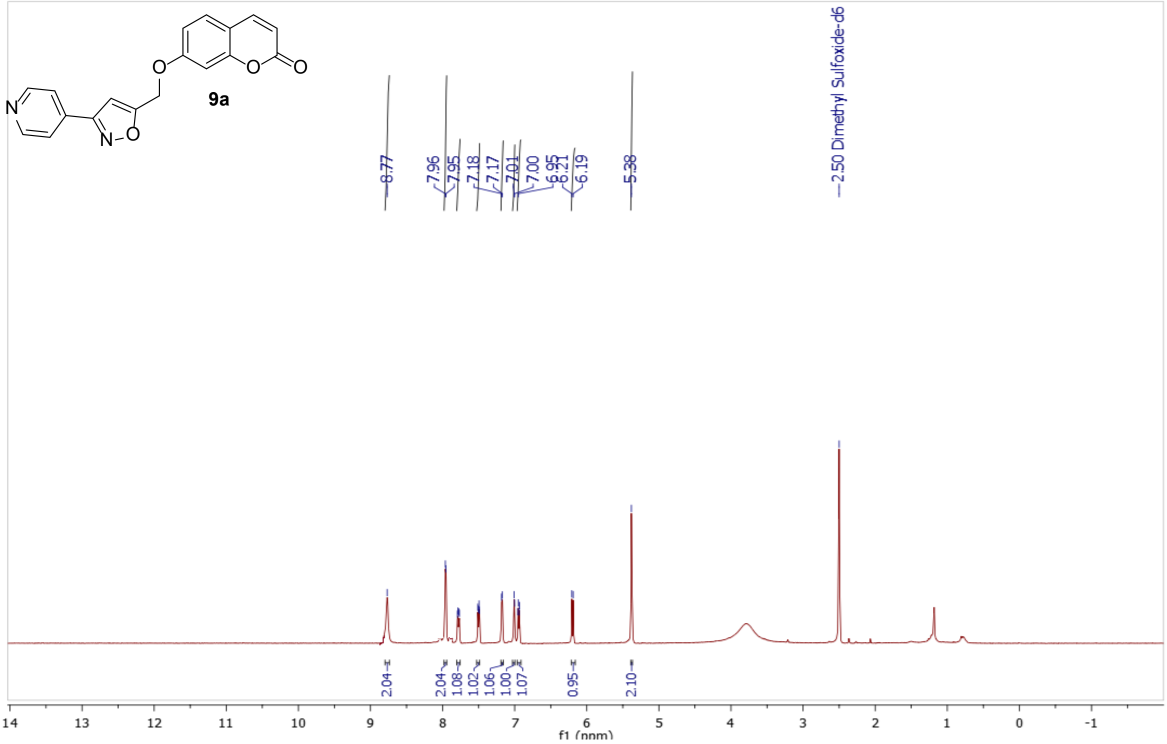

Figure S10. ^{1}H-NMR spectrum of **9a** (500 MHz, DMSO-d_6).

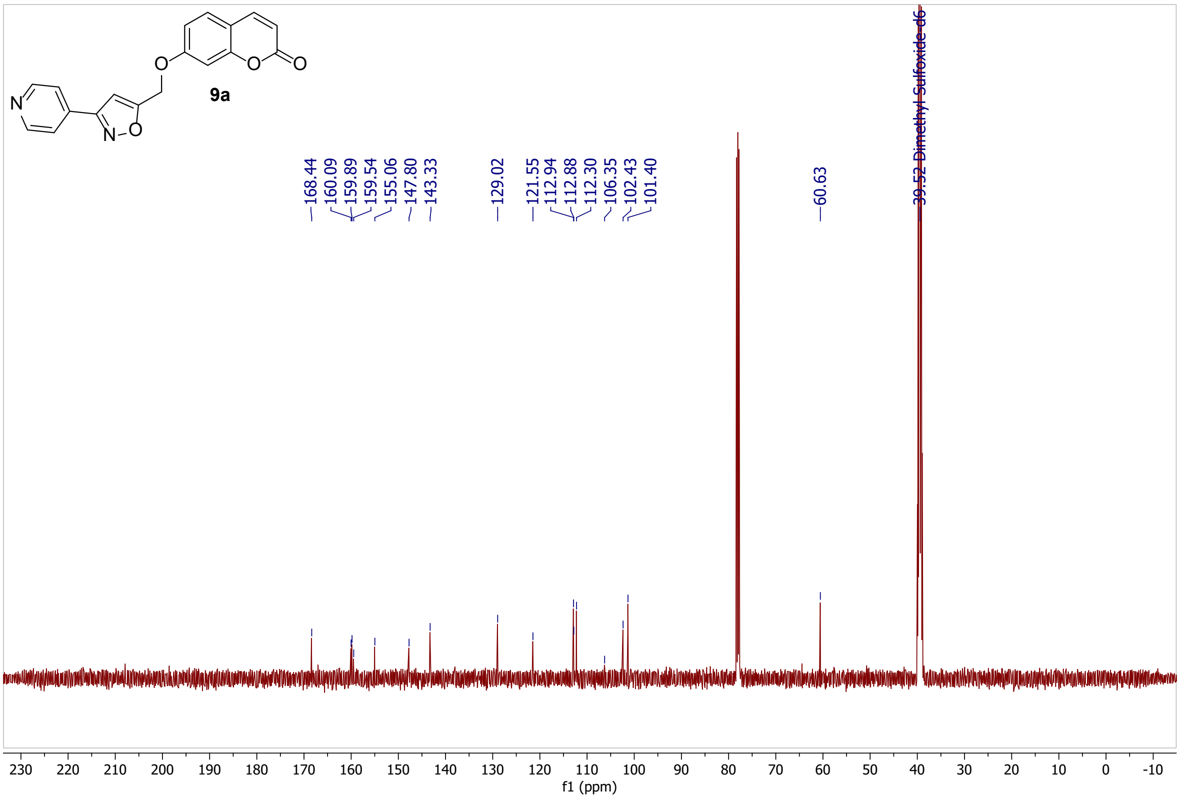

Figure S11. ^{13}C-NMR spectrum of **9a** (500 MHz, $CDCl_3$/DMSO-d_6).

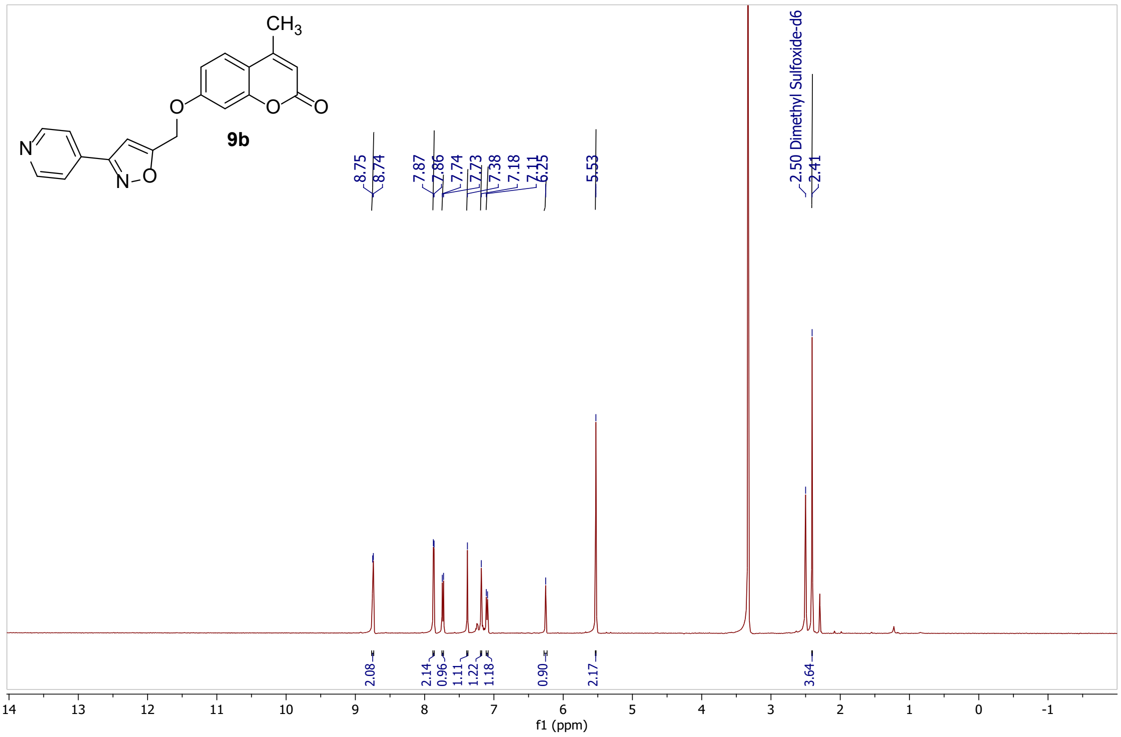

Figure S12. ^{1}H-NMR spectrum of **9b** (500 MHz, DMSO-d$_6$).

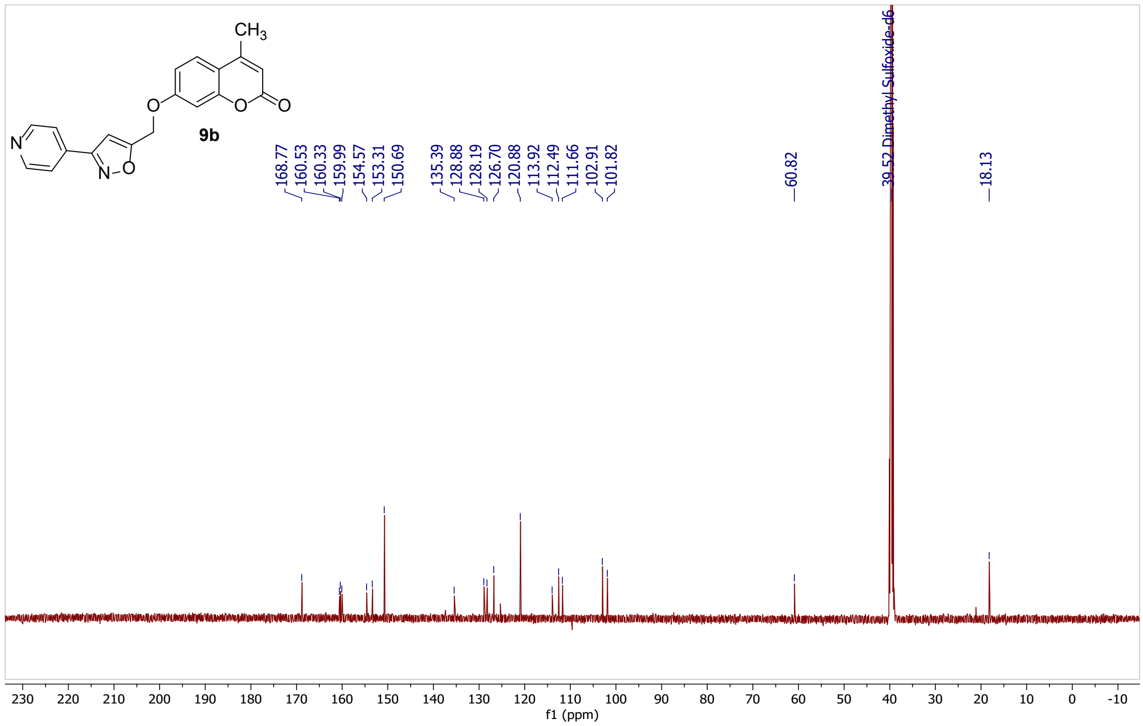

Figure S13. ^{13}C-NMR spectrum of **9b** (500 MHz, DMSO-d$_6$).

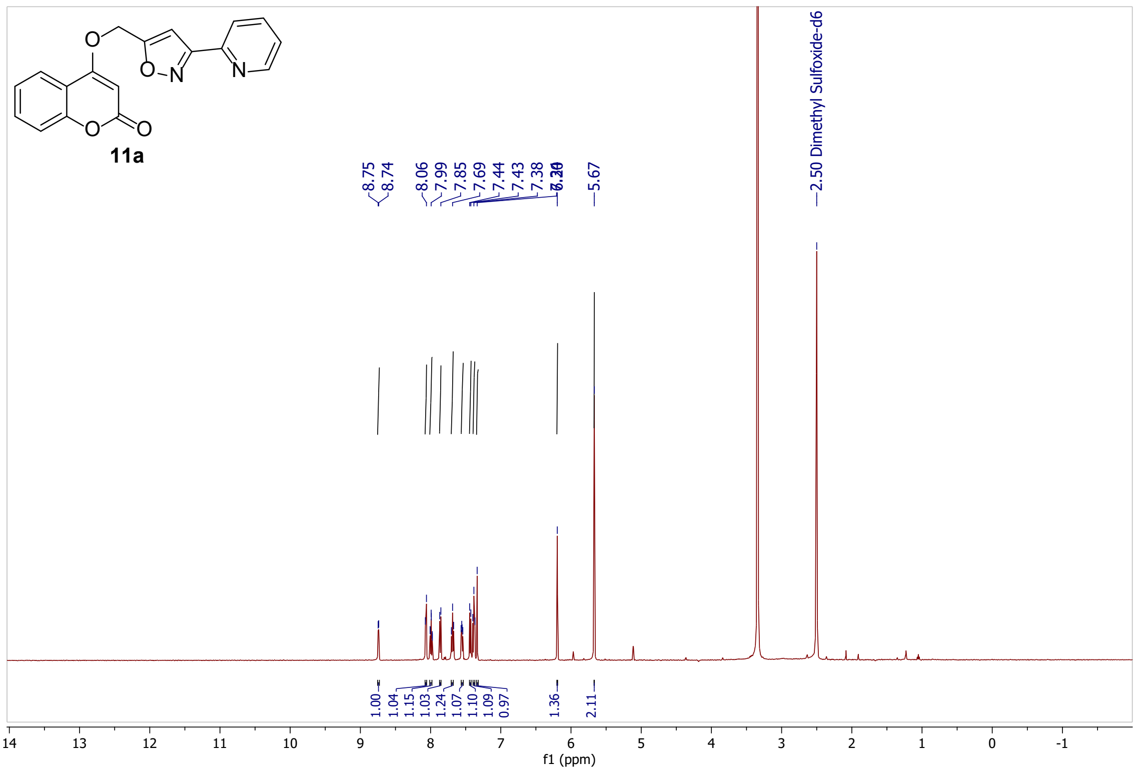

Figure S14. ^{1}H-NMR spectrum of **11a** (500 MHz, DMSO-d$_6$).

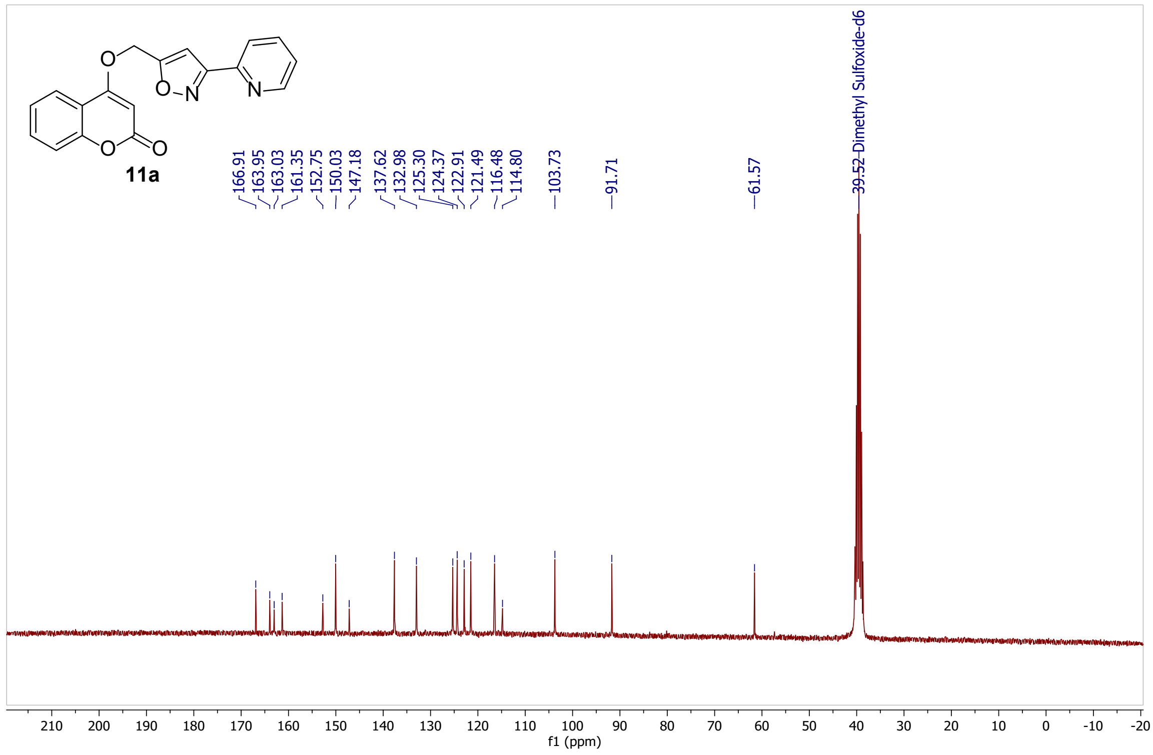

Figure S15. ^{13}C-NMR spectrum of **11a** (500 MHz, DMSO-d$_6$).

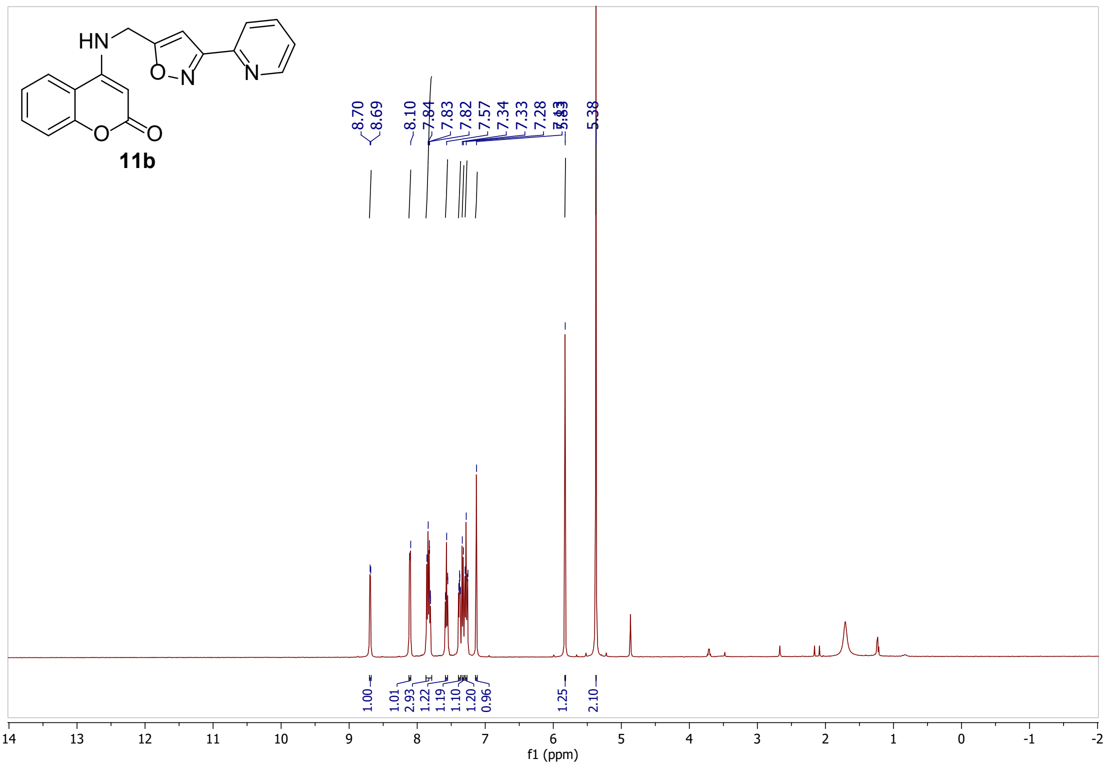

Figure S16. ^{1}H-NMR spectrum of **11b** (500 MHz, $CDCl_3$)

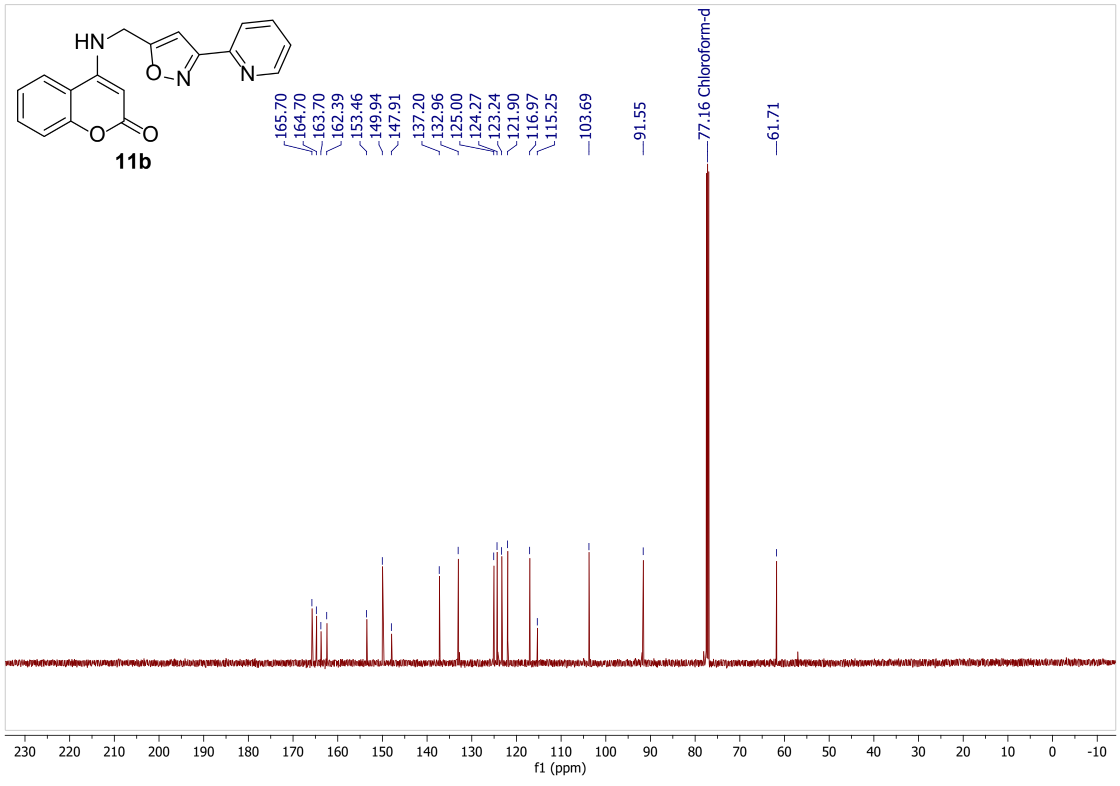

Figure S17. ^{13}C-NMR spectrum of **11b** (500 MHz, $CDCl_3$).

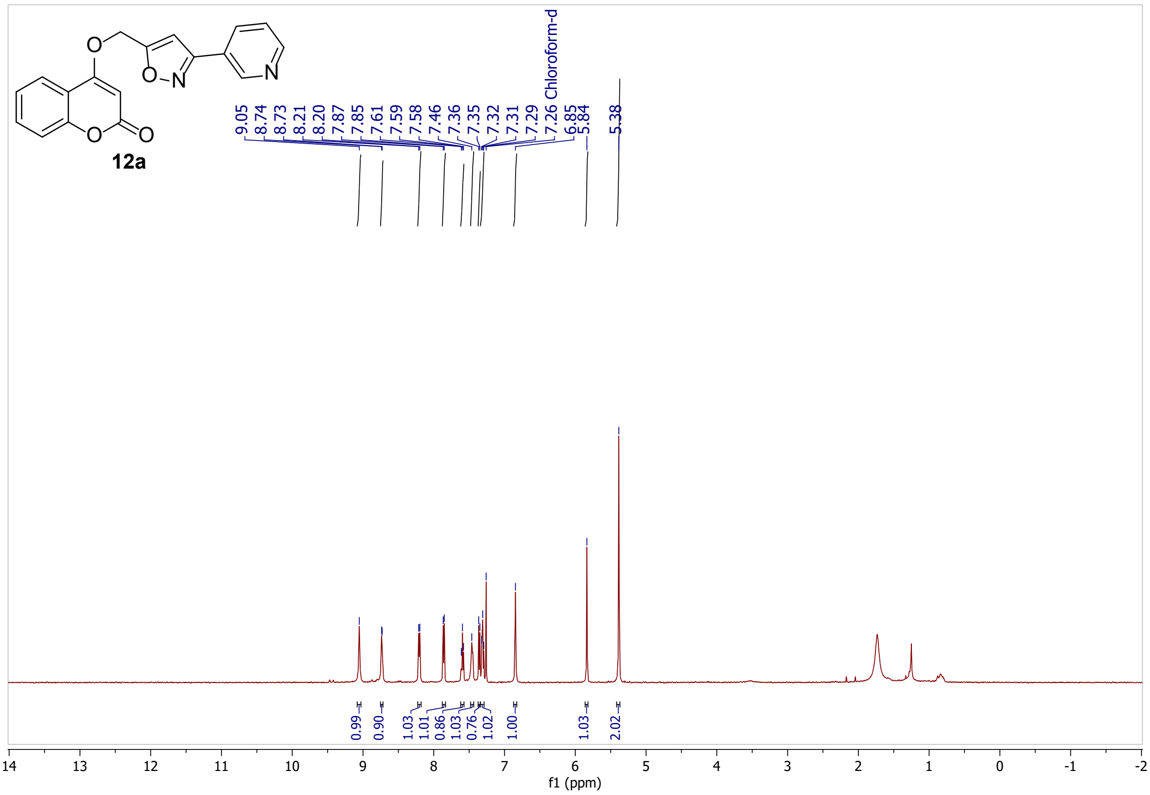

Figure S18. ^{1}H-NMR spectrum of **12a** (500 MHz, $CDCl_3$).

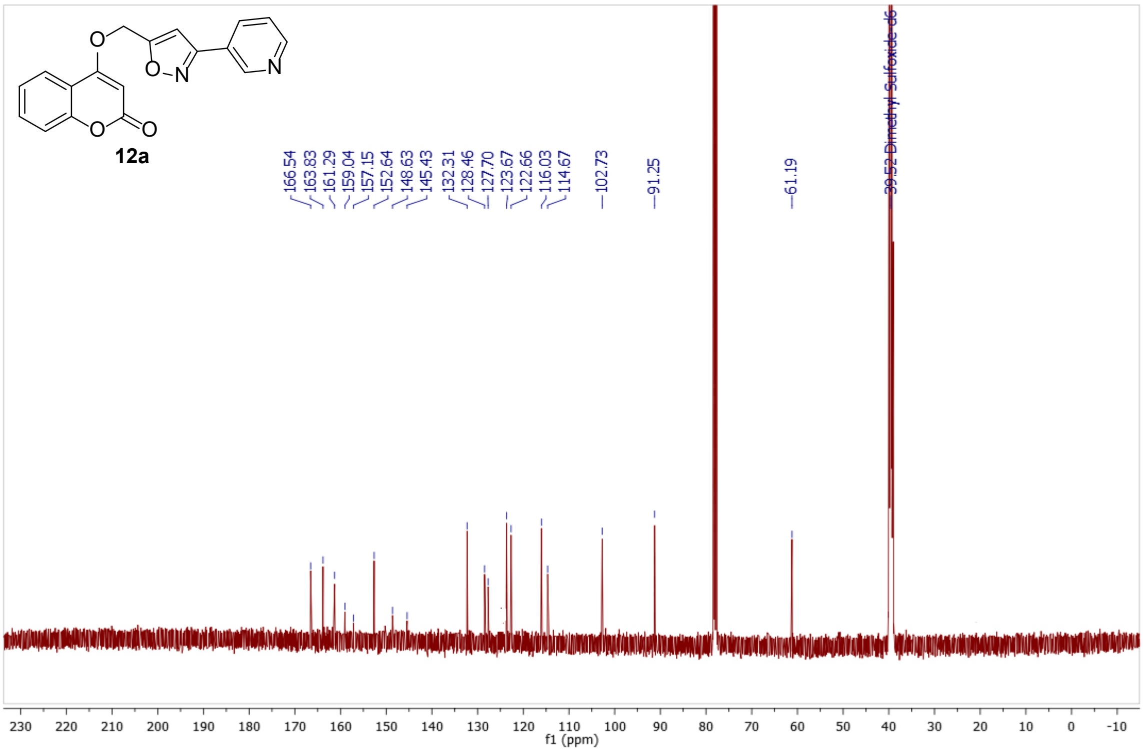

Figure S19. ^{1}H-NMR spectrum of **12a** (500 MHz, $CDCl_3$/DMSO-d_6).

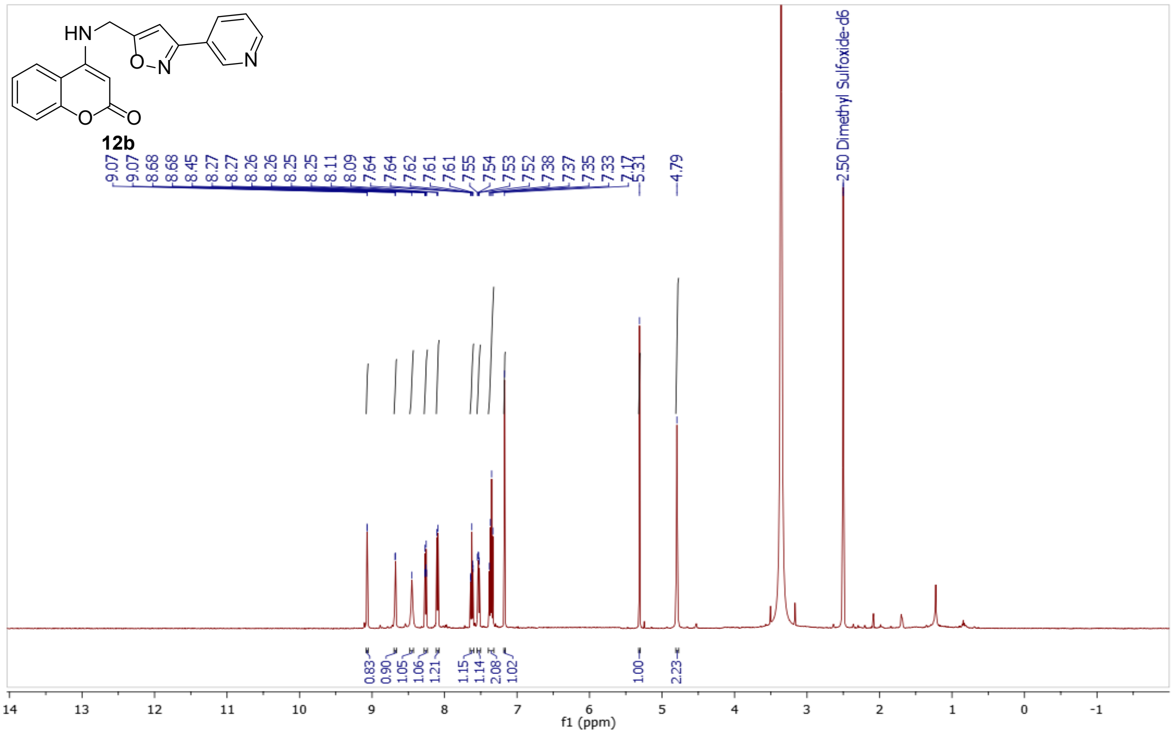

Figure S20. ^{1}H-NMR spectrum of **12b** (500 MHz, DMSO-d_6).

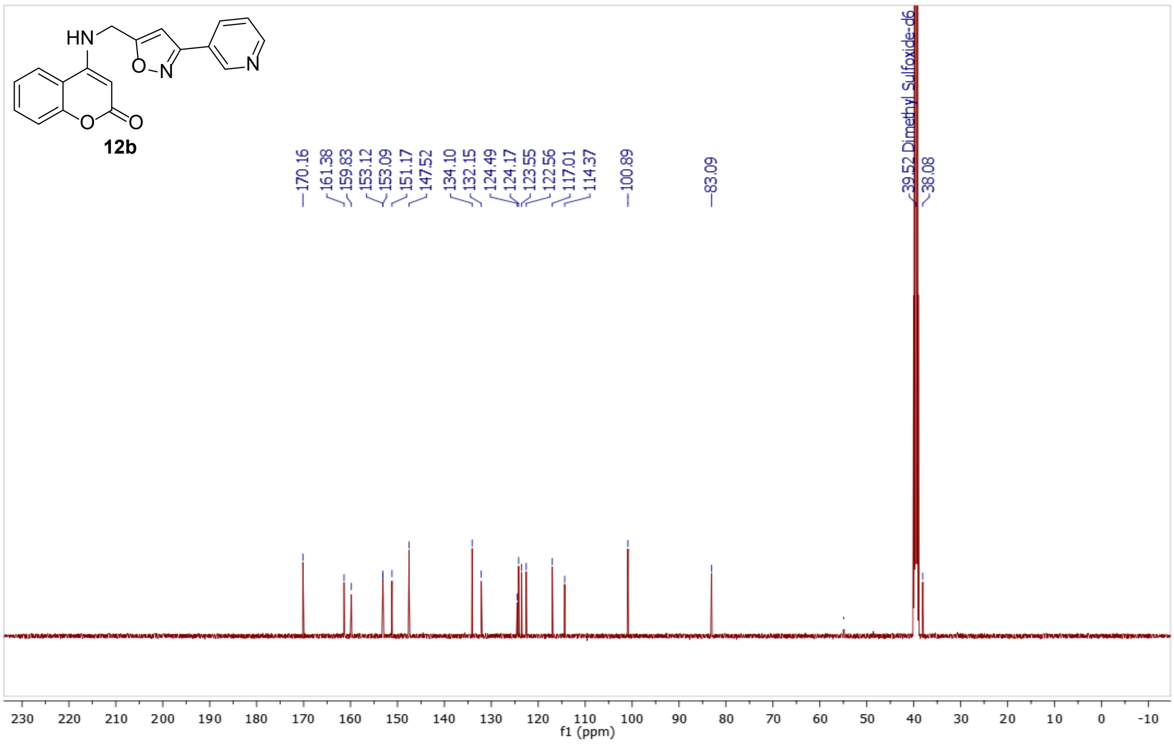

Figure S21. ^{13}C-NMR spectrum of **12b** (500 MHz, DMSO-d_6).

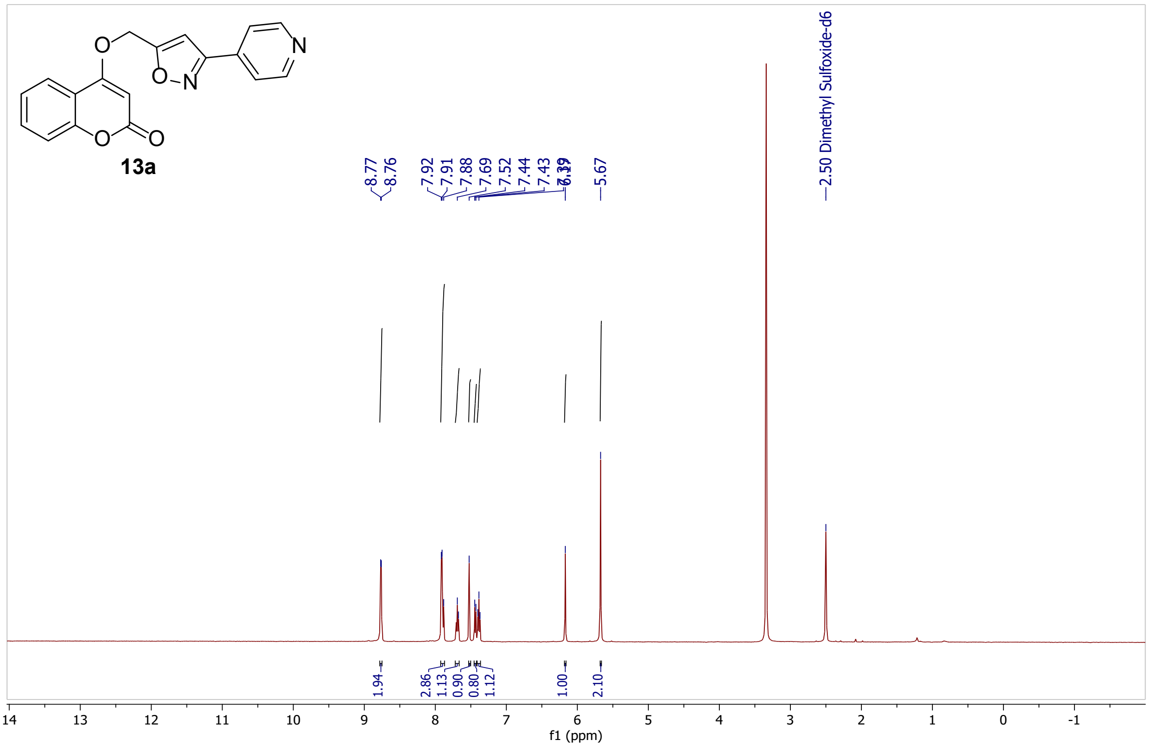

Figure S22. ^{1}H-NMR spectrum of **13a** (500 MHz, DMSO-d_6).

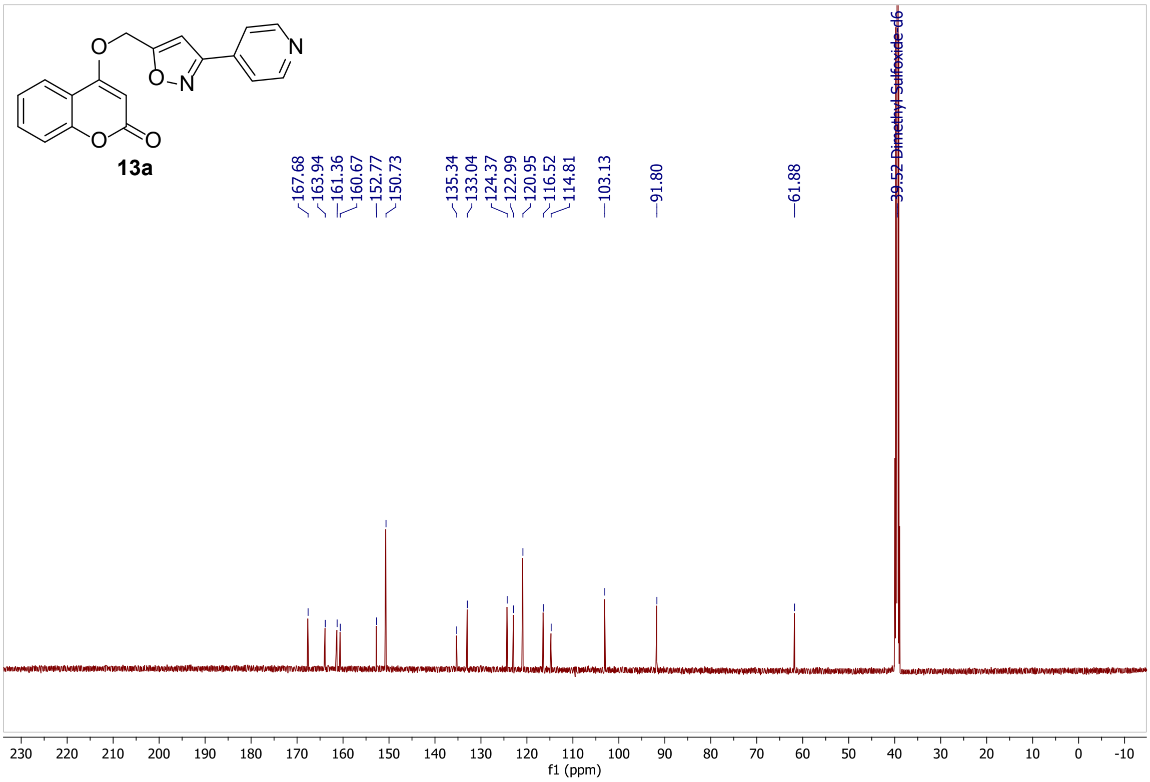

Figure S23. ^{13}C-NMR spectrum of **13a** (500 MHz, DMSO-d_6).

**Sample Name**	**3a**	**Position**	P2-A3	**Instrument Name**	Instrument 1
**User Name**		**Inj Vol**	15	**InjPosition**	
**Sample Type**	Sample	**IRM Calibration Status**	Success	**Data Filename**	MD541.d
**ACQ Method**	Fragmentor_Final_1500_B2ACNFormic.m	**Comment**		**Acquired Time**	10/25/2023 16:56:48

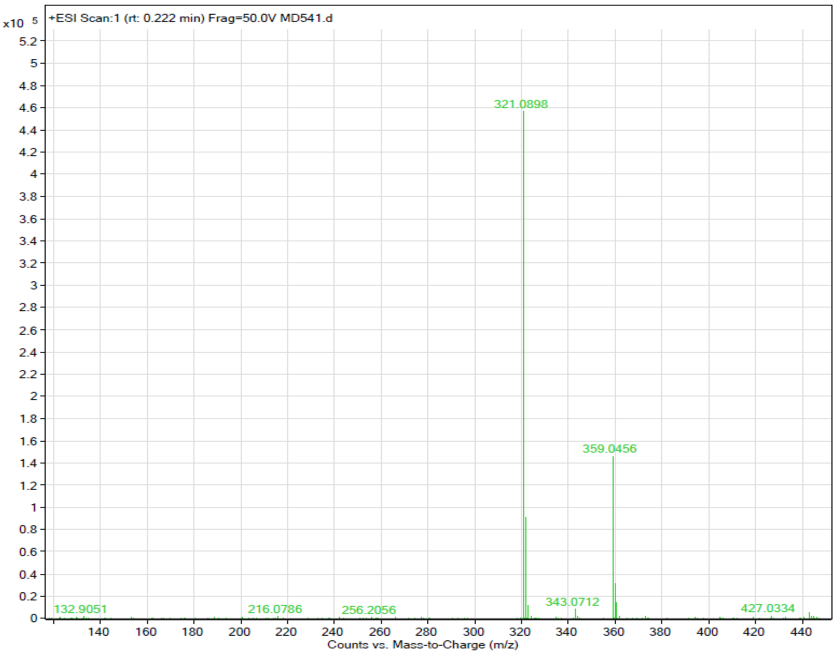

Figure S24. HRMS (ESI) of **3a**.

**Sample Name**	**3b**	**Position**	P2-A2	**Instrument Name**	Instrument 1
**User Name**		**Inj Vol**	15	**InjPosition**	
**Sample Type**	Sample	**IRM Calibration Status**	Success	**Data Filename**	MD549.d
**ACQ Method**	Fragmentor_Final_1500_B2ACNFormic.m	**Comment**		**Acquired Time**	10/25/2023 16:53:52

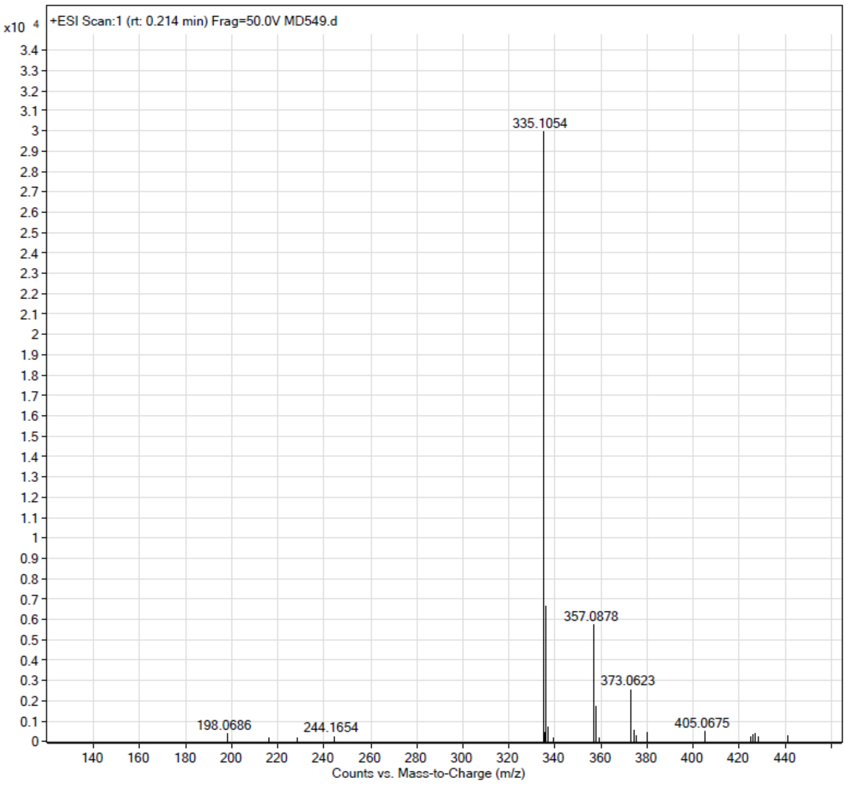

Figure S25. HRMS (ESI) of **3b**.

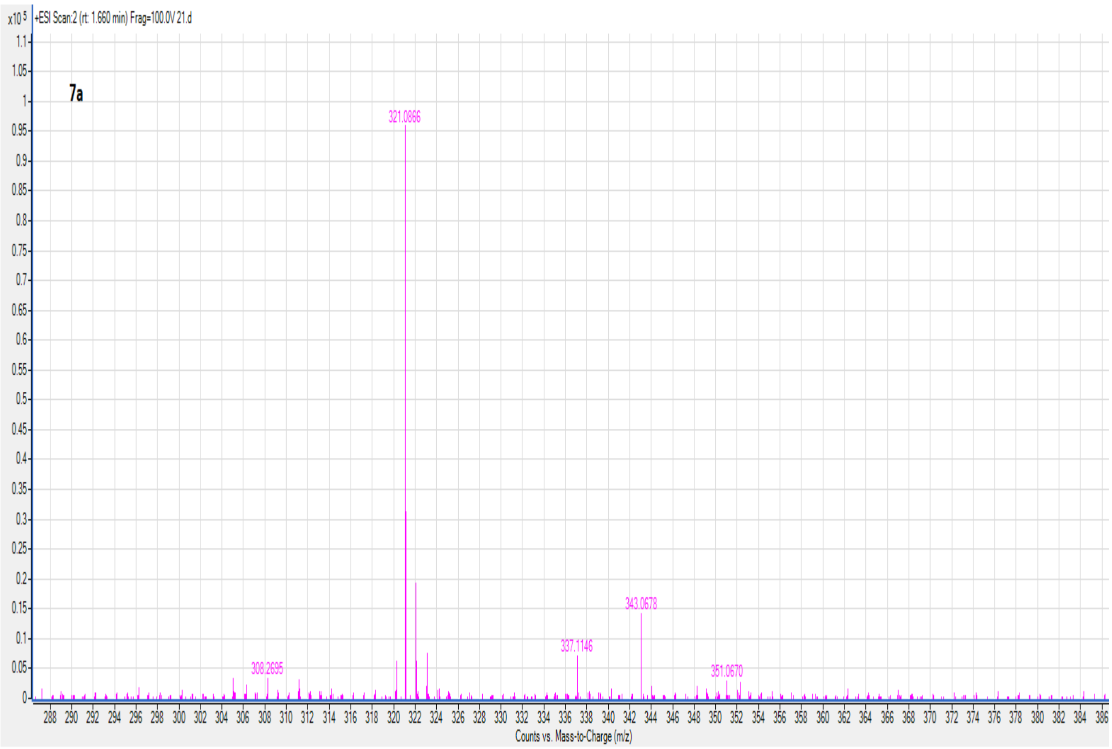

Figure S26. HRMS (ESI) of **7a**.

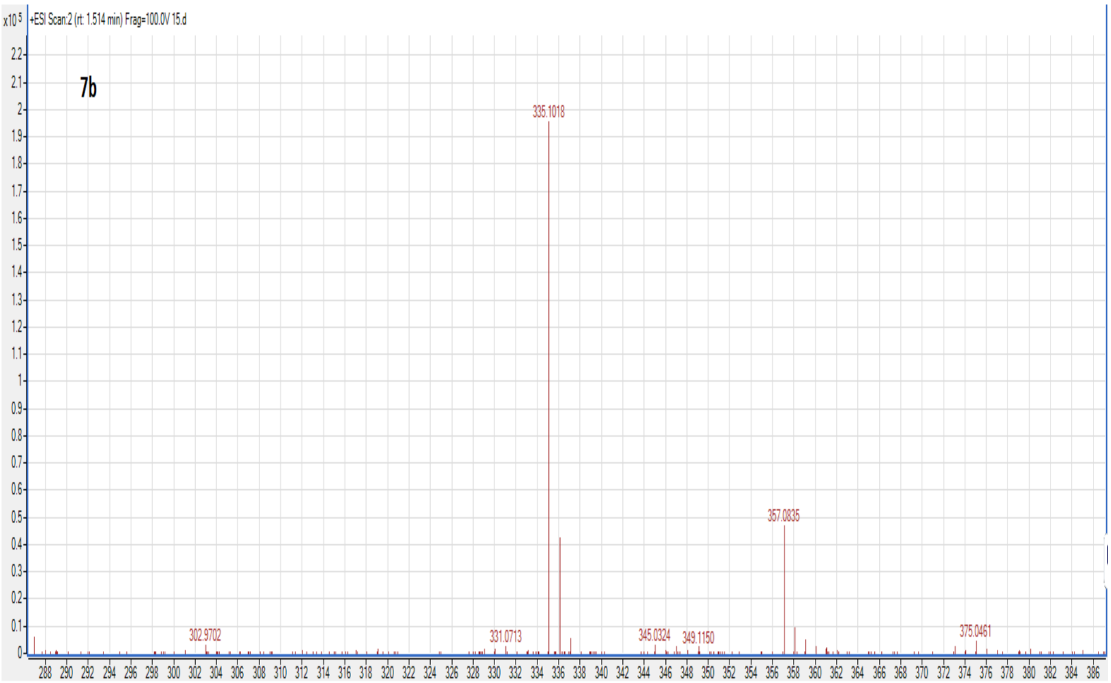

Figure S27. HRMS (ESI) of **7b**.

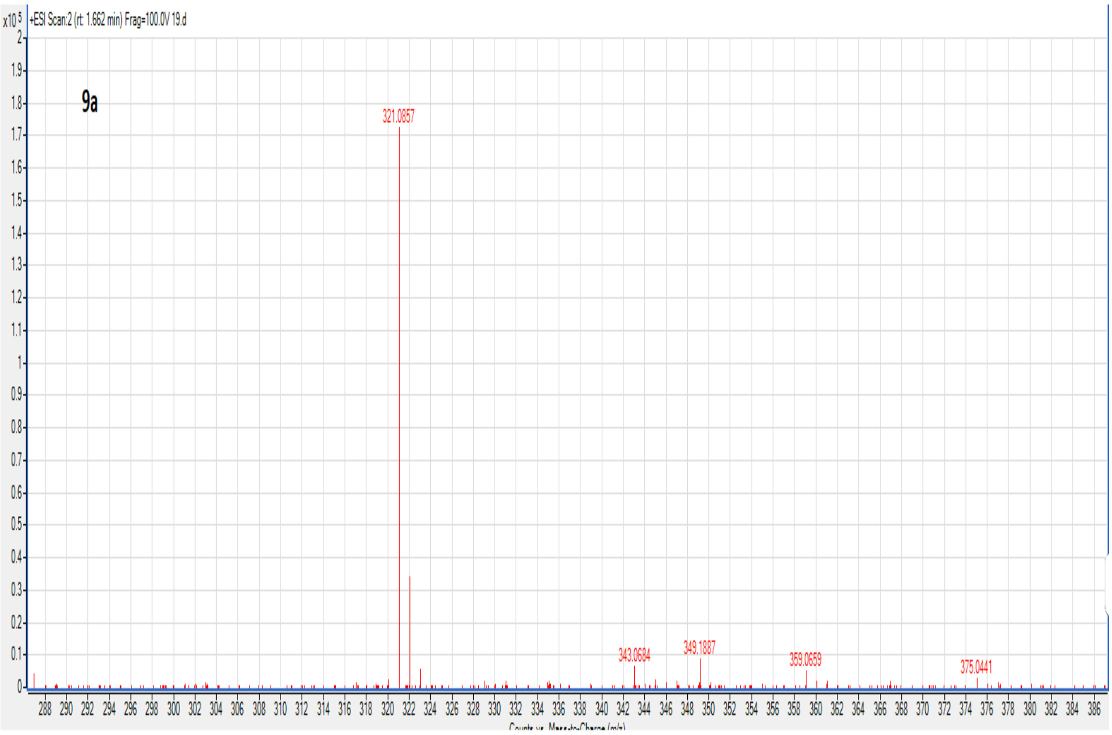

Figure S28. HRMS (ESI) of **9a**.

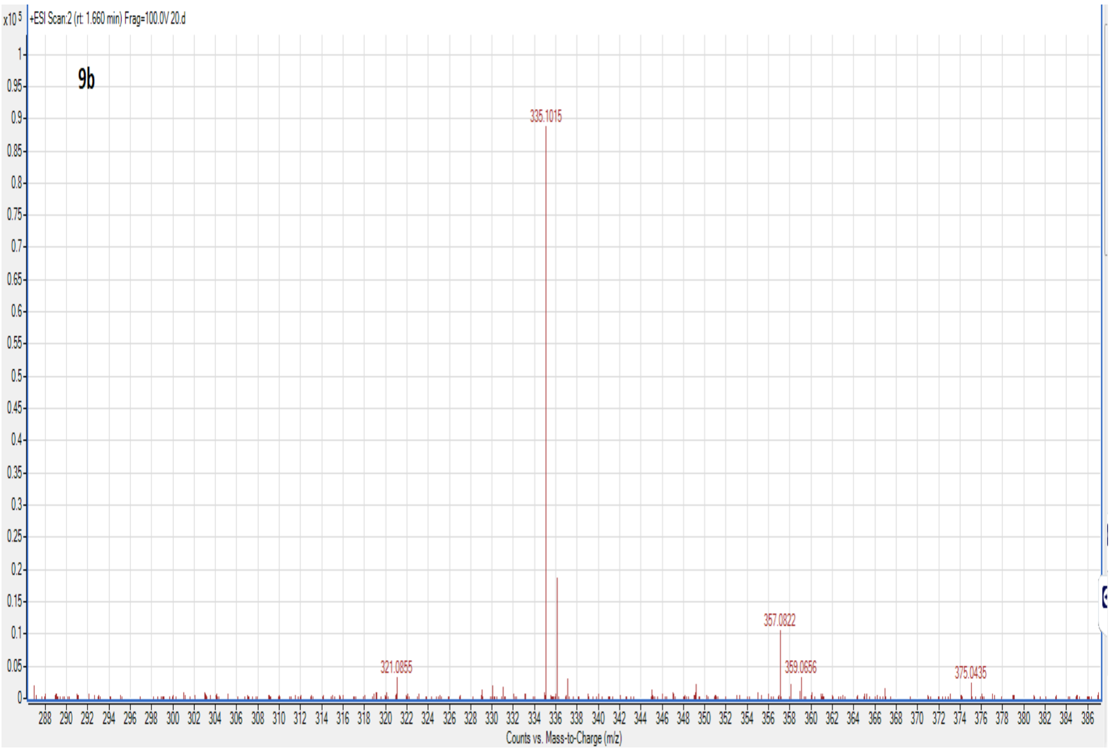

Figure S29. HRMS (ESI) of **9b**.

**Sample Name**	**11a**	**Position**	P1-F8	**Instrument Name**	Instrument 1
**User Name**		**Inj Vol**	15	**InjPosition**	
**Sample Type**	Sample	**IRM Calibration Status**	Success	**Data Filename**	MD538.d
**ACQ Method**	Fragmentor_Final_1500_B2ACNFormic.m	**Comment**		**Acquired Time**	10/25/2023 16:45:03

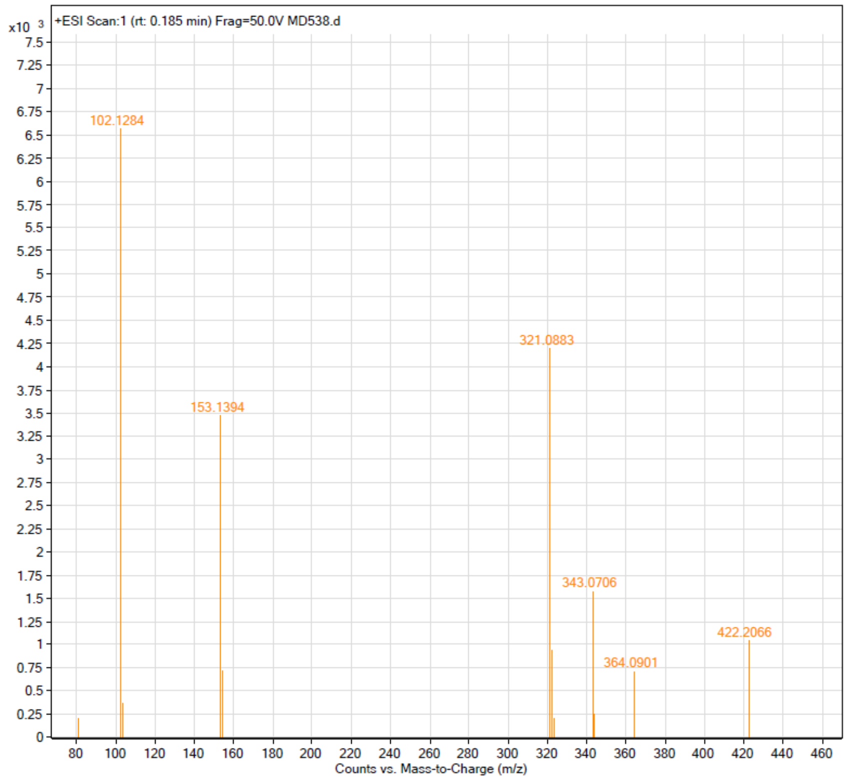

Figure S30. HRMS (ESI) of **11a**.

**Sample Name**	**11b**	**Position**	P1-F7	**Instrument Name**	Instrument 1
**User Name**		**Inj Vol**	15	**InjPosition**	
**Sample Type**	Sample	**IRM Calibration Status**	Success	**Data Filename**	MD519_solidB.d
**ACQ Method**	Fragmentor_Final_1500_B2ACNFormic.m	**Comment**		**Acquired Time**	10/25/2023 16:42:07

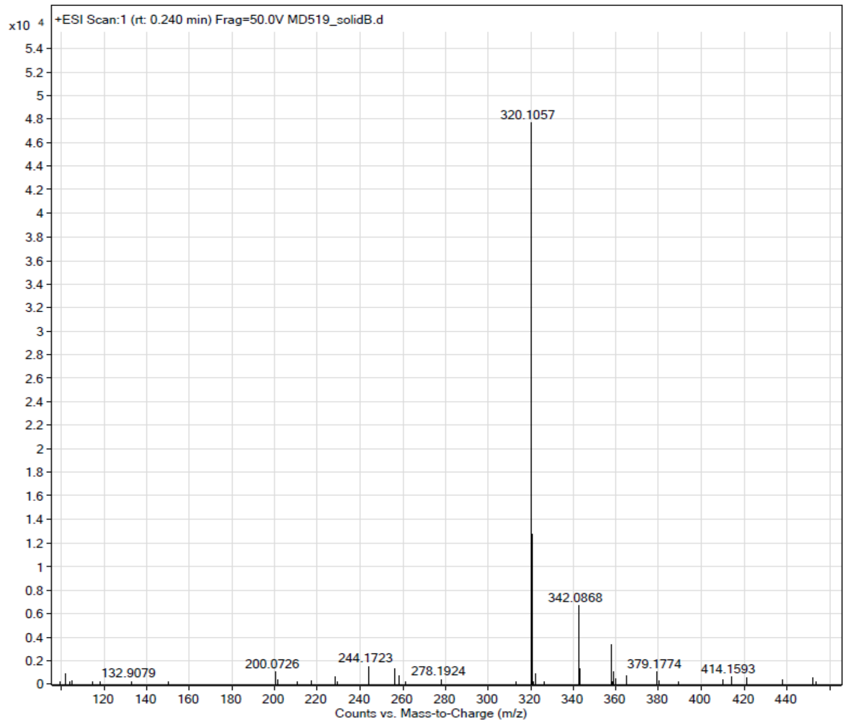

Figure S31. HRMS (ESI) of **11b**.

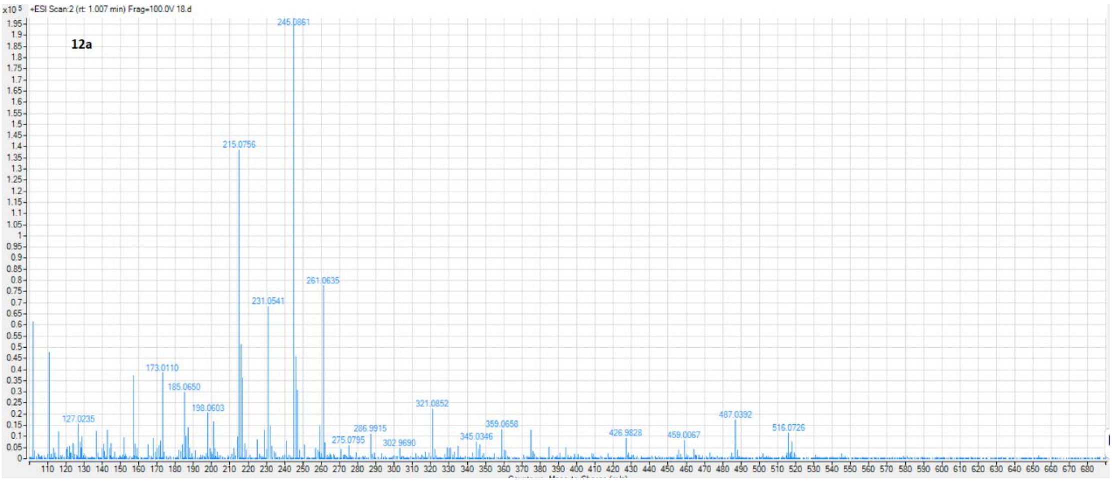

Figure S32. HRMS of **12a**.

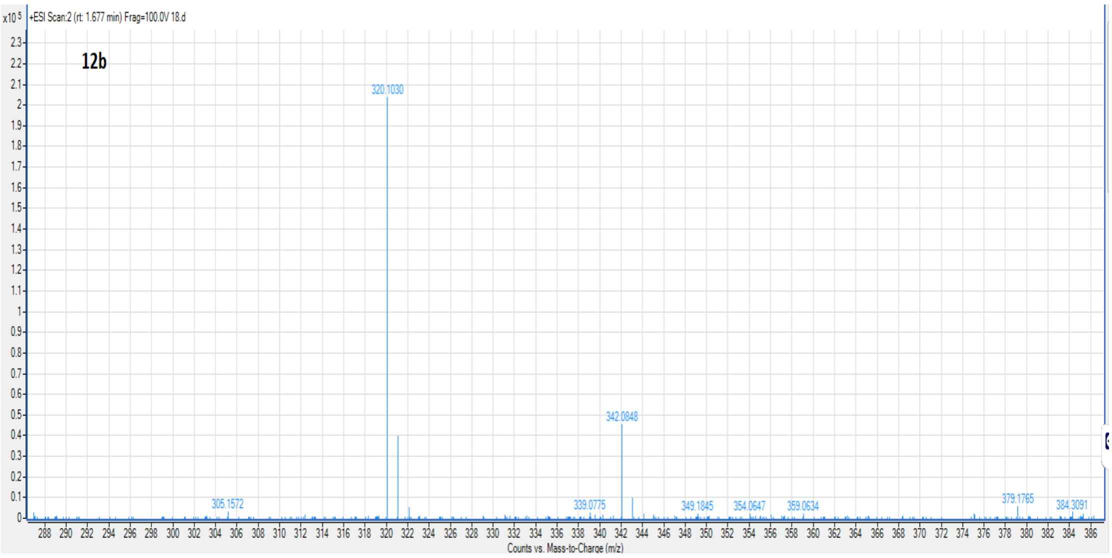

Figure S33. HRMS (ESI) of **12b**.

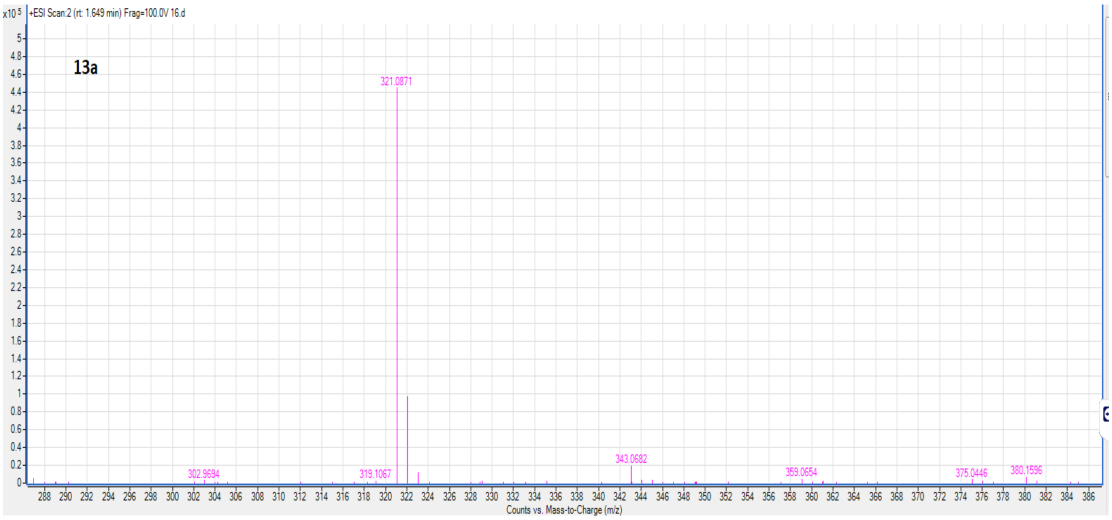

Figure S34. HRMS of **13a**.

Possible mechanistic Schemes for the dehydration of picoline aldehyde oxime (2)

DEHYDRATION with PIDA

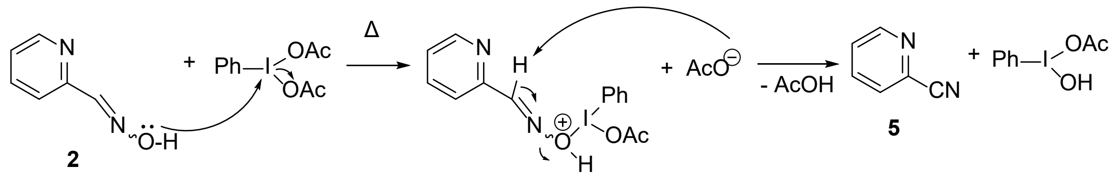

DEHYDRATION with TBN

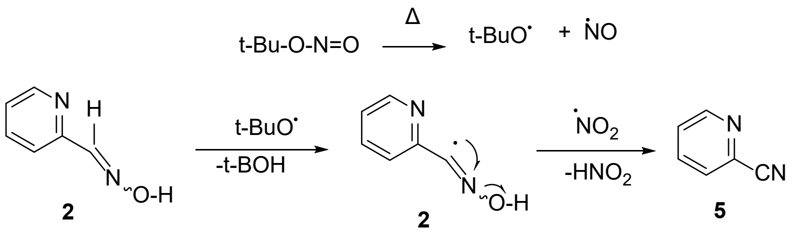

Supplement: Supplementary file 1 [file molecules-30-01592-s001.zip › molecules-3531810-supplementary.pdf]
